# The global, regional, and national brain and central nervous system cancer burden and trends from 1990 to 2021: an analysis based on the Global Burden of Disease Study 2021

**DOI:** 10.3389/fneur.2025.1574614

**Published:** 2025-06-18

**Authors:** Xueling Zhao, Menghao He, Renyi Yang, Nuojin Geng, Xinhua Zhu, Ning Tang

**Affiliations:** ^1^The First Hospital of Hunan University of Chinese Medicine, Changsha, Hunan, China; ^2^Hunan University of Chinese Medicine, Changsha, Hunan, China

**Keywords:** brain and central nervous system (CNS) cancer, global burden of disease, prevalence, incidence, mortality, disability-adjusted life years

## Abstract

**Background:**

Brain and central nervous system (CNS) cancers remain a significant contributor to mortality worldwide. This study aims to provide the latest assessment of the prevalence, incidence, mortality, and disability-adjusted life years (DALY) rates of brain and CNS cancers from 1990 to 2021 at the global, regional, and national levels, stratified by sex, age, and the Sociodemographic Index (SDI).

**Methods:**

Data from the Global Burden of Disease (GBD) database were used to analyze the age-standardized prevalence (ASPR), incidence (ASIR), mortality (ASDR), and DALY rates of brain and CNS cancers. Joinpoint regression was employed to calculate the annual percent change (APC), and a log-transformed linear regression model was used to estimate the average annual percent change (EAPC) for trend analysis. The data were stratified by sex, 20 age groups, 21 GBD regions, 204 countries/territories, and five SDI quintiles.

**Results:**

In 2021, there were an estimated 975,279.16 (95% UI, 857,199.67–1,096,203.50) global cases of brain and CNS cancers. The ASPR was 12.01 (95% UI, 10.54–13.52) per 100,000 population; the ASIR was 4.28 (95% UI, 3.71–4.88) per 100,000; the ASDR was 3.06 (95% UI, 2.62–3.50) per 100,000; and the age-standardized DALY rate was 49.58 (95% UI, 28.22–69.92) per 100,000. By SDI regions, the High SDI region showed the highest ASPR and ASIR, the High-Middle SDI region had the highest ASDR and age-standardized DALY rates, and the Low SDI region reported the lowest rates. Geographically, the High-income Asia Pacific region recorded the highest ASPR, Western Europe the highest ASIR, and Central Europe the highest ASDR and age-standardized DALY rates. Overall, in most regions globally, ASIR, ASDR, and age-standardized DALY rates among males increased with age and exceeded those of females. In high-SDI regions, the burden of brain and CNS cancers was predominantly in older adults, whereas in low-SDI regions, the burden among children was pronounced.

**Conclusion:**

The global burden of brain and CNS cancers is highest in High and High-Middle SDI regions, with a particularly severe burden in children in Low SDI regions. A comprehensive understanding of the epidemiology of brain and CNS cancers is crucial for strengthening disease prevention and control efforts worldwide.

## Introduction

Brain and central nervous system (CNS) cancer is highly lethal. Although Brain and CNS cancers are relatively rare worldwide (accounting for approximately 1.6% of all adult cancers) (1), they nonetheless contribute significantly to global morbidity and mortality. In 2016, there were 330,000 new cases of Brain and CNS cancer and 227,000 deaths worldwide, and incidence has continued to rise in recent years. From 1990 to 2016, the global age-standardized incidence rate (ASIR) of Brain and CNS cancer increased by 17.3% (2). The burden of Brain and CNS cancer continued to increase markedly by 2019 (3), and in 2020, an estimated 308,102 new cases and 251,329 deaths were reported globally (2). Recent research indicates that the incidence and mortality of Brain and CNS cancer in Asia are expected to rise over the next 25 years (especially among women) (4), and early-onset Brain and CNS cancer in young adults aged 20–49 may peak worldwide between 2035 and 2040 (5), posing a major challenge to global healthcare systems.

From an anatomical perspective, Brain and CNS cancer encompasses tumors of the brain, meninges, spinal cord, cranial nerves, other parts of the central nervous system, and tumors of the pituitary and pineal glands, as well as olfactory tumors in the nasal cavity. Due to the complex histology of CNS cancers, the World Health Organization’s tumor classification lists a wide range of types (6). A global systematic study of brain and CNS tumors observed that China and the United States accounted for the highest numbers of cases (2). In 2020, China reported 79,575 newly diagnosed cases of brain and CNS tumors and 65,204 related deaths. However, studies focusing on the burden of brain and CNS cancer remain scarce or incomplete (7). According to the latest statistical report by the Central Brain Tumor Registry of the United States (CBTRUS), between 2017 and 2021, about 27.1% of Brain and CNS cancers in the United States were malignant. Glioblastoma was the most common malignant CNS cancer, accounting for 14.0% of all CNS cancers and 51.5% of malignant CNS cancers. The remaining 72.9% of Brain and CNS cancers were benign, among which meningioma was the most common, representing 41.4% of all CNS cancers and 56.8% of non-malignant CNS cancers (8). Additionally, although childhood brain and CNS cancers are relatively rare (with an average annual age-adjusted incidence rate of 5.61 per 100,000), brain and other CNS tumors (both malignant and non-malignant) are the most common cancer site among children aged 0–14 in the United States, surpassing other cancers to become the leading cause of cancer deaths in this age group (8).

Primary malignant brain and CNS cancers have a poor prognosis and directly affect neurological function, commonly manifesting as headaches, vision loss, seizures, speech impairment, and paralysis (9). Glioblastoma is the most prevalent primary brain tumor. Although progress has been made in surgical resection, targeted radiotherapy, high-dose chemotherapy, and tumor treating fields, the invasive nature of these tumors, drug resistance, and the relatively immune-privileged status of the central nervous system significantly limit therapeutic efficacy. Consequently, clinical outcomes remain discouraging, with a median overall survival of only 14.6 to 20.5 months (10, 11), placing a considerable burden on society, families, and individuals. Several previous studies have focused on the burden of Brain and CNS cancers in Asia, the Middle East, and North Africa in 2019 (3, 4). More recently, another study provided an updated assessment of the burden of Brain and CNS cancers in men at the global, regional, and national levels from 1990 to 2021 (12). However, to date, no comprehensive analysis has been conducted on the global burden of Brain and CNS cancers using the latest data from the Global Burden of Disease Study 2021 (GBD 2021). With healthcare systems evolving, populations growing and aging, and persistent imbalances in regional development worldwide, it is essential to determine whether progress has been made in incidence, mortality, and survival. This will help identify the areas needing further investigation to address the ongoing challenges. Therefore, this study aims to provide the most recent assessment of global, regional, and national trends in the prevalence, incidence, mortality, and DALYs of Brain and CNS cancers from 1990 to 2021, thereby offering guidance for reducing the global burden of these diseases.

## Materials and methods

### Data sources and study population

The GBD 2021 results were derived from the Global Burden of Disease Collaborative Network, led by the Institute for Health Metrics and Evaluation (IHME) at the University of Washington. These results draw on the latest epidemiological data and rigorous standardized methods to provide a comprehensive assessment of health losses globally, across five SDI levels and 21 regions, 204 countries, 369 diseases, injuries, and health-related conditions, as well as 88 risk factors (13). The estimates were generated through the standardized GBD 2021 methodology, including Bayesian meta-regression using DisMod-MR 2.1. Data sources were cross-checked against national vital registration systems, cancer registries, and WHO databases where applicable.

In this study, we obtained and analyzed GBD data pertaining to Brain and CNS cancer at the global, regional, and national levels, stratified by sex (male, female) and age (19 age groups from <5 years to ≥95 years in 5-year increments). Specifically, we examined the age-standardized prevalence rate (ASPR), incidence rate (ASIR), mortality rate (ASDR), and disability-adjusted life years (DALY) rate, as well as the absolute numbers for each measure. These data were accessed through the GBD results tool.^1^ All estimates are presented with 95% uncertainty intervals (95% UIs) or 95% confidence intervals (95% CIs). The 95% UIs for each measure were defined by the 2.5th and 97.5th percentiles of 1,000 draws from the posterior distribution. Detailed information on the study design and methods of the GBD project has been extensively described in existing GBD literature. Furthermore, the University of Washington Institutional Review Board waived the requirement for informed consent to access GBD data. This study used publicly available data and adhered to the Guidelines for Accurate and Transparent Health Estimates Reporting (GATHER).

### Sociodemographic index

The Sociodemographic Index (SDI) is a composite measure of a country’s or region’s socioeconomic development. It typically incorporates factors such as income level, educational attainment, health status, poverty level, and overall quality of life, which are then weighted and combined into a single index value. This value reflects the overall level of social development. The SDI ranges from 0 to 1, with higher values generally indicating higher levels of social development and lower values suggesting deficiencies in social development. The SDI is typically categorized into five groups: low (<0.46), low-middle (0.46–0.60), middle (0.61–0.69), high-middle (0.70–0.81), and high (>0.81). This classification aids in examining the impact of socioeconomic indicators and geographical disparities on the burden of Brain and CNS cancers.

### Statistical analysis

Using data from the GBD database, we calculated trends in the age-standardized rates (ASR) of prevalence, incidence, mortality, and DALYs per 100,000 population for Brain and CNS cancer. A log-transformed linear regression model was applied to compute the estimated annual percent change (EAPC) for describing the ASR trends in the burden of Brain and CNS cancer. If the EAPC and the lower limit of its 95% confidence interval (CI) were both greater than zero, the ASR was considered to be increasing; conversely, if the EAPC and the upper limit of its 95% CI were both below zero, the ASR was considered to be decreasing. If neither condition was met, the ASR was considered stable. A Joinpoint regression model was used to calculate the average annual percent change (AAPC) and its 95% CI to evaluate trends within each discrete time period. EAPC focuses on measuring the rate of change over a single time interval (thus identifying significant upward or downward trends irrespective of short-term fluctuations), whereas AAPC serves as a weighted average of the annual changes over multiple time intervals, summarizing the overall trend throughout the study period (14). We employed decomposition analysis to attribute differences in incidence, mortality, and DALYs of Brain and CNS cancer from 1990 to 2021 to three independent factors: aging, population changes, and epidemiological changes. The decomposition results present both the absolute and relative contributions of each factor to changes in incidence, mortality, or DALYs (15). Additionally, the concentration index of inequality (CII) and the slope index of inequality (SII) were used to quantify socioeconomic, geographic, and demographic disparities in the burden of Brain and CNS cancer, thereby revealing the key drivers of health inequalities (16). Furthermore, in this study, we used a Bayesian age-period-cohort (BAPC) model with integrated nested Laplace approximation (INLA) to predict future trends in the burden of Brain and CNS cancer (17). All analyses were conducted using R version 4.4.2 and Jingding statistical software, with a significance level of *p* < 0.05.

## Results

### Global level

**Figure 1:**
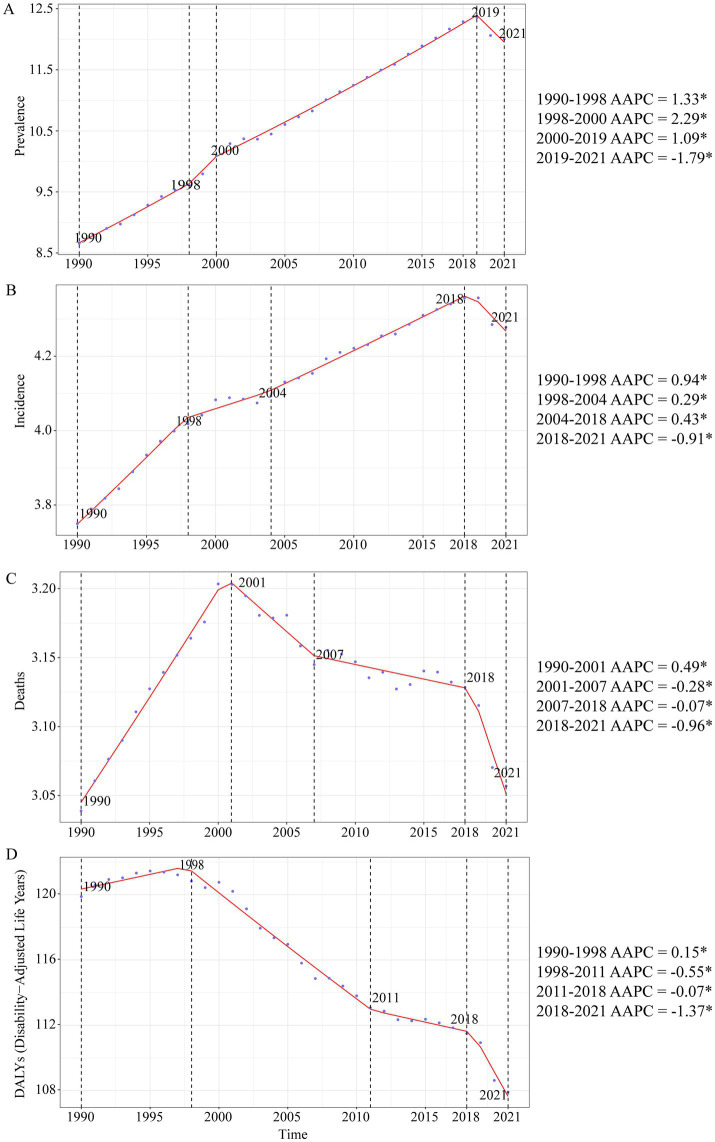
Annual average percent change (AAPC) and trends in global Brain and CNS cancer from 1990 to 2021 for prevalence, incidence, deaths, and disability-adjusted life years (DALYs). **(A)** ASPR; **(B)** ASIR; **(C)** ASDR; **(D)** Age standardized-DALYs rate.

**Figure 2:**
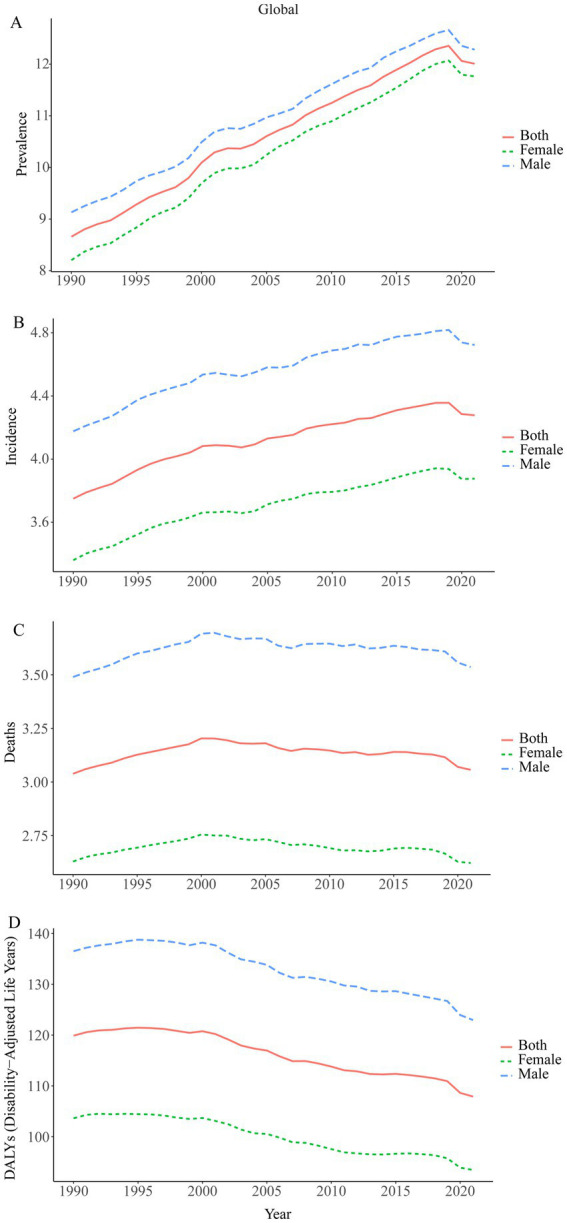
Time trends in the age-standardized prevalence, incidence, deaths, and disability-adjusted life years (DALYs) of Brain and CNS cancer from 1990 to 2021. **(A)** ASPR; **(B)** ASIR; **(C)** ASDR; **(D)** Age standardized-DALYs rate.

A large-scale data analysis in this study indicates that the burden of Brain and CNS cancer has been consistently rising, with only a slight slowdown in recent years. In 2021, the global number of Brain and CNS cancer cases was 975,279.16 (95% UI, 857,199.67–1,096,203.50), compared to 434,423.55 (95% UI, 375,266.41–480,677.82) in 1990, reflecting a remarkable overall increase of 124.50% (95% UI, 98.46–149.94). From 1998 to 2000, the highest AAPC (average annual percent change) in ASPR (age-standardized prevalence rate) was observed, at 2.29% (95% CI, 1.63–2.96%). The ASPR rose from 8.66 (95% UI, 7.55–9.53) per 100,000 population in 1990 to 12.01 (95% UI, 10.54–13.52) per 100,000 in 2021, with an EAPC (estimated annual percent change) of 1.48% (95% CI, 1.43–1.53%). In 2021, there were 357,482.30 (95% UI, 310,456.68–407,432.57) new Brain and CNS cancer cases worldwide, an increase of 106.53% compared with 173,086.44 (95% UI, 147,452.47–194,951.22) in 1990. The highest AAPC in ASIR (age-standardized incidence rate) was recorded from 1990 to 1998, at 0.94% (95% CI, 0.85–1.03%). The ASIR increased from 3.75 (95% UI, 3.21–4.21) per 100,000 population in 1990 to a peak of 4.28 (95% UI, 3.71–4.88) per 100,000 in 2021, resulting in an EAPC of 1.11% (95% CI, 1.08–1.14%). Approximately 258,626.80 (95% UI, 222,185.21–296,133.94) deaths were attributable to Brain and CNS cancer worldwide in 2021, up 89.86% from 136,218.86 (95% UI, 114,799.30–155,197.44) in 1990. Between 1990 and 2001, the highest AAPC in ASDR (age-standardized mortality rate) was 0.49% (95% CI, 0.46–0.53%). Mortality rates changed only slightly over the period, from 3.04 (95% UI, 2.58–3.45) per 100,000 population in 1990 to 3.06 (95% UI, 2.62–3.50) per 100,000 in 2021, yielding an EAPC of 0.80% (95% CI, 0.78–0.82%). The age-standardized DALY rate related to Brain and CNS cancer has generally shown a downward trend since 1998. In 2021, there were 8,912,595.21 (95% UI, 7,612,510.59–10,356,061.06) DALYs globally. From 1990 to 1998, the highest AAPC was 0.15% (95% CI, 0.05–0.25%). The age-standardized DALY rate decreased from 119.88 (95% UI, 99.23–137.57) per 100,000 population in 1990 to 49.58 (95% UI, 28.22–69.92) per 100,000 in 2021, with an EAPC of 0.01% (95% CI, −0.03–0.04%). Overall, the ASPR and ASIR for Brain and CNS cancer have been increasing globally, only beginning to show a decline around 2019, whereas the ASDR and age-standardized DALY rate have been declining since around 2000. These trends may be associated with ongoing developments in population demographics, diagnostic and therapeutic technologies, and public health measures. Regarding sex-specific patterns, the trends in males and females parallel the overall trends; however, between 1990 and 2020, males displayed higher ASPR, ASIR, ASDR, and age-standardized DALY rates than females (Table 1; Figures 1, 2).

**Table 1:** Global and regional trends in brain and CNS cancer burden: prevalence, incidence, mortality, and disability-adjusted life years (1990–2021).

Location	1990		2021		1990–2021		
	Number ASR		Number ASR		Number change ASR change EAPC		
Prevalence							
Global	434423.55(375266.41–480677.82)	8.66(7.55, 9.53)	975279.16(857199.67–1096203.50)	12.01(10.54, 13.52)	124.50(98.46–149.94)	38.66(23.03, 53.00)	1.48(1.43–1.53)
High SDI	171073.98(166987.05–174514.59)	18.73(18.29, 19.09)	346210.68(325277.68–361913.90)	26.10(24.84, 27.19)	102.37(92.93–110.95)	39.35(34.36, 44.90)	1.72(1.62–1.83)
High-middle SDI	112569.36(95513.13–125819.57)	10.77(9.12, 12.05)	257158.92(222678.71–301841.62)	18.58(16.02, 21.83)	128.44(97.25–166.33)	72.46(48.71, 101.26)	2.19(2.12–2.27)
Middle SDI	106422.41(79187.97–125164.98)	6.45(4.85, 7.55)	261399.49(212241.15–313333.05)	10.49(8.54, 12.53)	145.62(99.47–200.70)	62.70(32.80, 95.47)	1.86(1.77–1.95)
Low-middle SDI	33958.25(24928.34–47174.82)	3.04(2.24, 4.17)	83321.38(65697.36–102461.91)	4.49(3.56, 5.54)	145.36(79.99–197.92)	47.43(12.60, 79.31)	1.40(1.35–1.44)
Low SDI	9977.21(6299.92–16397.15)	2.03(1.28, 3.09)	26499.66(18495.16–33547.10)	2.53(1.76, 3.24)	165.60(81.66–236.74)	24.81(−7.49, 52.55)	0.64(0.51–0.78)
Andean Latin America	1889.63(1494.46–2687.43)	5.15(4.10, 7.15)	6628.73(5278.28–8522.15)	10.09(8.04, 12.93)	250.80(138.01–372.61)	95.75(37.41, 159.61)	2.61(2.32–2.90)
Australasia	3378.51(3234.02–3545.19)	16.24(15.47, 17.07)	5862.98(5409.66–6358.98)	16.79(15.47, 18.28)	73.54(58.66–90.61)	3.41(−5.78, 14.60)	0.68(0.42–0.94)
Caribbean	2110.07(1921.21–2717.28)	6.26(5.76, 7.90)	4145.06(3633.33–4833.90)	8.44(7.38, 9.89)	96.44(68.87–125.93)	34.81(18.59, 53.81)	1.58(1.46–1.71)
Central Asia	4330.27(3683.75–5037.49)	6.30(5.29, 7.25)	9909.35(8623.00–11323.82)	10.14(8.84, 11.57)	128.84(85.72–178.54)	61.02(31.01, 97.72)	1.87(1.71–2.04)
Central Europe	14736.72(14044.14–15811.20)	11.35(10.81, 12.18)	19673.97(17907.94–21578.09)	14.50(13.14, 15.96)	33.50(21.01–45.22)	27.70(15.49, 38.62)	1.38(1.04–1.73)
Central Latin America	6765.25(6525.48–7056.56)	4.33(4.21, 4.47)	16655.68(14743.39–19026.16)	6.54(5.78, 7.50)	146.19(115.99–179.72)	51.06(32.88, 71.89)	1.51(1.30–1.71)
Central Sub-Saharan Africa	583.20(398.33–906.28)	1.25(0.91, 1.69)	1753.88(1200.81–2305.98)	1.60(1.07, 2.17)	200.73(85.55–315.41)	28.00(−13.12, 67.92)	0.79(0.56–1.02)
East Asia	111497.57(80212.65–134751.60)	9.63(6.94, 11.67)	309958.80(249101.36–392236.52)	20.83(16.70, 26.00)	178.00(120.75–263.13)	116.34(71.86, 175.98)	2.89(2.79–2.98)
Eastern Europe	17078.99(15781.74–18134.38)	7.28(6.78, 7.69)	23311.44(21277.25–25421.54)	9.80(9.05, 10.62)	36.49(20.35–57.50)	34.59(20.55, 52.83)	1.36(1.23–1.50)
Eastern Sub-Saharan Africa	4263.84(2916.69–6696.05)	2.15(1.45, 3.04)	11485.00(8483.81–15062.91)	2.81(2.04, 3.60)	169.36(73.48–258.19)	30.86(−9.76, 61.40)	0.79(0.63–0.96)
High-income Asia Pacific	30597.19(28293.12–32256.65)	17.07(15.78, 18.04)	102110.95(88331.04–112325.92)	36.44(32.00, 39.83)	233.73(201.33–265.10)	113.53(93.92, 131.86)	4.08(3.82–4.34)
High-income North America	65462.55(63887.06–67019.13)	22.77(22.26, 23.29)	103233.70(98839.36–107432.77)	25.21(24.20, 26.19)	57.70(51.32–64.18)	10.69(5.96, 15.20)	0.65(0.54–0.76)
North Africa and Middle East	23486.69(16814.99–33086.58)	7.16(5.33, 10.14)	64887.16(46722.75–82234.31)	10.82(7.81, 13.69)	176.27(73.27–238.73)	51.11(−1.87, 81.23)	1.51(1.40–1.63)
Oceania	71.31(35.74–97.07)	1.19(0.60, 1.59)	175.07(93.92–232.23)	1.32(0.70, 1.76)	145.50(102.47–194.92)	10.90(−7.59, 32.99)	0.44(0.36–0.53)
South Asia	29335.70(18720.97–39545.01)	2.83(1.84, 3.80)	65612.74(53185.66–88062.33)	3.64(2.94, 4.89)	123.66(62.80–221.79)	28.81(−1.95, 85.17)	0.83(0.68–0.98)
Southeast Asia	15012.58(10512.08–18684.81)	3.48(2.46, 4.28)	33885.47(24754.69–40529.20)	4.79(3.49, 5.71)	125.71(67.86–172.34)	37.77(4.88, 65.28)	1.35(1.30–1.39)
Southern Latin America	3259.57(2930.09–3619.27)	6.66(5.99, 7.39)	7473.59(6867.84–8066.94)	10.72(9.78, 11.66)	129.28(96.59–165.32)	60.87(37.96, 86.50)	2.21(1.89–2.54)
Southern Sub-Saharan Africa	1139.15(888.23–1494.37)	2.57(2.00, 3.28)	2543.24(1944.23–3139.05)	3.38(2.57, 4.13)	123.26(80.19–160.94)	31.60(9.99, 54.59)	1.27(1.16–1.38)
Tropical Latin America	10728.54(10100.89–11323.90)	7.66(7.23, 8.09)	26559.05(25425.10–27607.54)	11.08(10.56, 11.58)	147.56(131.40–166.05)	44.64(35.46, 54.75)	1.76(1.56–1.95)
Western Europe	86786.02(84647.21–88761.92)	21.13(20.64, 21.63)	152922.98(145878.11–158855.41)	28.90(27.78, 29.95)	76.21(68.21–83.85)	36.75(30.68, 42.48)	1.60(1.35–1.86)
Western Sub-Saharan Africa	1910.21(1261.92–2534.10)	0.86(0.59, 1.09)	6490.33(3261.32–8417.43)	1.28(0.67, 1.64)	239.77(99.05–333.14)	48.07(−8.29, 87.49)	1.23(1.04–1.41)
Incidence							
Global	173086.44(147452.47–194951.22)	3.75(3.21, 4.21)	357482.30(310456.68–407432.57)	4.28(3.71, 4.88)	106.53(82.94–129.34)	14.09(1.68, 26.59)	1.11(1.08–1.14)
High SDI	55694.12(54173.05–56847.27)	5.66(5.52, 5.78)	100993.68(94963.67–105628.62)	6.38(6.08, 6.64)	81.34(73.62–87.91)	12.62(9.25, 16.58)	1.25(1.19–1.31)
High-middle SDI	49615.13(42241.78–55310.85)	4.81(4.10, 5.35)	97818.17(83423.12–112504.75)	5.90(5.05, 6.77)	97.15(71.38–126.07)	22.62(6.24, 39.82)	1.59(1.56–1.62)
Middle SDI	47057.61(35595.13–56516.65)	3.35(2.57, 4.07)	106918.22(86956.26–128833.12)	4.11(3.34, 4.94)	127.21(88.12–174.23)	22.65(2.21, 46.87)	1.55(1.50–1.60)
Low-middle SDI	15846.92(11647.73–21805.63)	1.73(1.31, 2.32)	39604.13(31857.51–49413.71)	2.34(1.89, 2.93)	149.92(92.36–202.56)	34.88(7.89, 64.89)	1.41(1.40–1.43)
Low SDI	4669.47(2949.14–7298.29)	1.23(0.77, 1.75)	11810.39(8189.76–15144.36)	1.43(1.00, 1.82)	152.93(84.39–208.17)	16.69(−6.97, 42.74)	0.41(0.29–0.54)
Andean Latin America	838.33(665.07–1161.40)	2.76(2.20, 3.73)	2793.67(2221.97–3479.50)	4.43(3.52, 5.50)	233.24(141.35–342.20)	60.23(17.77, 110.67)	2.36(2.05–2.66)
Australasia	1393.20(1343.26–1447.54)	6.32(6.10, 6.56)	2536.34(2341.92–2729.75)	5.95(5.54, 6.38)	82.05(68.93–97.10)	−5.82(−12.33, 1.80)	0.62(0.53–0.71)
Caribbean	896.55(823.71–1130.73)	2.93(2.72, 3.61)	1971.17(1725.14–2269.86)	3.85(3.37, 4.46)	119.86(90.16–148.86)	31.39(16.62, 48.14)	1.97(1.86–2.08)
Central Asia	1965.95(1639.67–2244.45)	3.24(2.68, 3.69)	4635.68(4012.80–5300.49)	4.94(4.29, 5.62)	135.80(92.47–191.33)	52.19(24.73, 88.68)	1.93(1.80–2.06)
Central Europe	8003.42(7657.03–8532.38)	5.76(5.50, 6.15)	11720.65(10689.02–12787.13)	6.64(6.07, 7.25)	46.45(34.85–57.29)	15.41(6.31, 24.36)	1.62(1.39–1.86)
Central Latin America	2825.21(2739.98–2916.07)	2.23(2.17, 2.29)	7585.80(6775.25–8533.38)	2.98(2.66, 3.36)	168.50(138.42–201.23)	33.94(19.42, 50.13)	1.63(1.42–1.84)
Central Sub-Saharan Africa	294.70(213.23–419.46)	0.83(0.60, 1.07)	876.40(586.26–1181.94)	1.03(0.67, 1.39)	197.39(92.76–299.32)	24.28(−12.40, 60.52)	0.69(0.50–0.88)
East Asia	48577.77(35371.79–60360.49)	4.63(3.39, 5.77)	107613.78(83225.28–135767.79)	6.02(4.69, 7.53)	121.53(73.79–182.37)	29.98(3.42, 62.73)	2.00(1.93–2.07)
Eastern Europe	9101.42(8399.80–9678.27)	3.63(3.37, 3.85)	14293.12(13133.92–15494.25)	4.98(4.60, 5.38)	57.04(39.01–79.93)	36.90(21.95, 55.11)	1.70(1.60–1.81)
Eastern Sub-Saharan Africa	1847.68(1233.90–2724.86)	1.25(0.81, 1.67)	4820.69(3456.72–6278.22)	1.53(1.05, 1.92)	160.91(72.31–230.44)	22.23(−10.39, 47.77)	0.62(0.50–0.74)
High-income Asia Pacific	5648.89(5036.86–5978.06)	3.11(2.76, 3.29)	16367.65(14108.19–18000.79)	5.44(4.76, 5.92)	189.75(158.80–215.64)	75.13(60.78, 89.78)	3.46(3.14–3.78)
High-income North America	22169.59(21557.53–22637.46)	7.11(6.93, 7.24)	36461.77(34376.30–37685.94)	7.08(6.75, 7.31)	64.47(58.58–69.06)	−0.30(−3.35, 2.38)	0.74(0.66–0.81)
North Africa and Middle East	10151.00(7655.94–14314.71)	4.09(3.16, 5.72)	26565.65(19733.69–32478.58)	4.98(3.70, 6.10)	161.70(74.13–215.10)	21.61(−17.08, 48.74)	1.32(1.23–1.41)
Oceania	32.44(16.14–43.49)	0.69(0.35, 0.94)	82.43(42.76–109.56)	0.74(0.38, 0.99)	154.09(107.66–205.90)	7.62(−10.39, 28.72)	0.58(0.50–0.66)
South Asia	13984.85(9077.78–18699.09)	1.61(1.05, 2.12)	31817.26(26008.92–43157.13)	1.87(1.53, 2.55)	127.51(72.57–232.13)	16.13(−7.95, 68.95)	0.84(0.70–0.97)
Southeast Asia	7050.98(5029.09–8698.73)	1.97(1.43, 2.45)	16690.80(12169.79–19949.90)	2.40(1.76, 2.87)	136.72(83.43–184.33)	21.96(−2.24, 47.93)	1.50(1.44–1.56)
Southern Latin America	1485.49(1349.82–1622.37)	3.12(2.84, 3.41)	2981.41(2776.22–3172.72)	3.81(3.55, 4.06)	100.70(76.95–126.25)	22.05(7.78, 37.47)	1.74(1.45–2.03)
Southern Sub-Saharan Africa	569.10(441.13–728.86)	1.57(1.20, 1.98)	1390.09(1024.05–1668.24)	2.05(1.50, 2.43)	144.26(105.17–184.86)	30.46(11.82, 53.01)	1.57(1.48–1.65)
Tropical Latin America	4966.58(4716.16–5251.44)	4.14(3.93, 4.40)	14100.91(13433.54–14672.84)	5.65(5.37, 5.88)	183.92(165.04–201.84)	36.44(27.69, 44.71)	2.17(1.98–2.37)
Western Europe	30521.26(29739.83–31118.08)	6.55(6.40, 6.66)	49619.44(46606.04–51778.61)	7.44(7.13, 7.71)	62.57(55.47–68.32)	13.62(9.70, 17.52)	1.23(1.11–1.34)
Western Sub-Saharan Africa	762.05(514.84–997.27)	0.43(0.30, 0.53)	2557.60(1314.70–3276.16)	0.63(0.34, 0.79)	235.62(103.13–322.88)	47.59(−5.26, 84.89)	1.16(1.02–1.30)
Deaths							
Global	136218.86(114799.30–155197.44)	3.04(2.58, 3.45)	258626.80(222185.21–296133.94)	3.06(2.62, 3.50)	89.86(67.34–112.95)	0.60(−10.55, 12.10)	0.80(0.78–0.82)
High SDI	37797.86(36602.98–38634.01)	3.69(3.57, 3.76)	63268.40(59449.36–66057.96)	3.54(3.36, 3.68)	67.39(60.82–73.03)	−4.08(−7.27, −1.06)	0.94(0.89–1.00)
High-middle SDI	40528.56(34406.72–45442.25)	3.96(3.36, 4.44)	71069.55(60266.03–81226.27)	3.94(3.36, 4.51)	75.36(53.75–100.87)	−0.48(−13.16, 13.64)	1.15(1.12–1.18)
Middle SDI	39417.54(30141.16–47589.45)	2.97(2.31, 3.63)	79778.83(64859.27–96616.88)	3.02(2.45, 3.65)	102.39(68.84–143.56)	1.72(−14.11, 20.41)	1.13(1.10–1.16)
Low-middle SDI	14060.86(10404.40–19411.61)	1.62(1.23, 2.18)	33995.99(27307.83–42716.74)	2.08(1.68, 2.62)	141.78(87.92–193.56)	28.10(3.86, 57.00)	1.30(1.28–1.32)
Low SDI	4240.21(2670.35–6640.61)	1.18(0.74, 1.68)	10224.58(7079.69–13086.13)	1.33(0.93, 1.69)	141.13(76.56–192.58)	12.90(−9.58, 37.91)	0.25(0.14–0.36)
Andean Latin America	721.03(573.40–985.58)	2.51(2.00, 3.36)	2198.55(1751.24–2725.11)	3.55(2.83, 4.40)	204.92(120.18–299.21)	41.35(4.11, 85.80)	2.06(1.75–2.37)
Australasia	1096.22(1059.91–1132.43)	4.85(4.69, 5.01)	1959.44(1798.71–2120.53)	4.21(3.90, 4.52)	78.75(65.50–92.91)	−13.24(−18.94, −6.75)	0.49(0.40–0.57)
Caribbean	727.65(665.71–935.66)	2.46(2.26, 3.05)	1633.52(1423.50–1901.08)	3.16(2.76, 3.68)	124.49(92.17–154.77)	28.56(13.71, 45.02)	2.08(1.96–2.20)
Central Asia	1724.80(1430.60–1966.52)	2.94(2.42, 3.35)	3999.11(3463.60–4565.71)	4.34(3.77, 4.92)	131.86(90.93–187.61)	47.52(20.60, 84.06)	1.85(1.72–1.97)
Central Europe	7156.67(6848.07–7639.06)	5.05(4.83, 5.40)	10772.30(9819.99–11747.80)	5.57(5.09, 6.08)	50.52(38.66–61.42)	10.16(1.23, 17.91)	1.72(1.53–1.92)
Central Latin America	2331.63(2267.42–2402.93)	1.97(1.92, 2.03)	6166.87(5505.30–6892.12)	2.44(2.17, 2.72)	164.49(136.09–195.54)	23.61(10.68, 37.99)	1.56(1.36–1.76)
Central Sub-Saharan Africa	271.60(198.80–380.81)	0.81(0.58, 1.05)	786.26(526.08–1070.85)	0.99(0.65, 1.35)	189.49(88.88–288.86)	22.31(−13.57, 59.19)	0.60(0.43–0.76)
East Asia	40077.38(29283.48–50222.73)	3.99(2.94, 4.96)	70564.81(53460.34–90081.86)	3.59(2.72, 4.53)	76.07(36.64–125.07)	−9.95(−29.17, 13.83)	1.13(1.07–1.19)
Eastern Europe	7940.80(7359.69–8418.77)	3.11(2.90, 3.29)	12717.48(11709.20–13757.60)	4.17(3.86, 4.50)	60.15(42.98–81.81)	33.96(19.99, 51.52)	1.73(1.63–1.83)
Eastern Sub-Saharan Africa	1652.56(1096.45–2426.90)	1.19(0.76, 1.57)	4120.50(2903.38–5341.33)	1.41(0.94, 1.77)	149.34(63.49–212.78)	18.30(−13.05, 43.26)	0.47(0.37–0.58)
High-income Asia Pacific	2332.10(1967.38–2478.81)	1.25(1.05, 1.33)	4905.63(4244.79–5347.68)	1.51(1.31, 1.63)	110.35(88.72–131.83)	21.02(11.61, 32.05)	2.23(1.96–2.50)
High-income North America	14577.94(14099.49–14871.48)	4.46(4.33, 4.54)	23966.65(22465.84–24781.42)	4.09(3.87, 4.22)	64.40(58.67–68.78)	−8.19(−10.89, −5.99)	0.74(0.63–0.85)
North Africa and Middle East	8735.17(6654.09–12267.16)	3.91(3.03, 5.52)	22279.74(16570.40–27228.32)	4.44(3.31, 5.43)	155.06(75.25–210.71)	13.58(−21.22, 41.76)	1.24(1.13–1.36)
Oceania	27.82(13.63–37.69)	0.65(0.34, 0.88)	71.35(37.28–95.89)	0.69(0.36, 0.92)	156.47(109.75–209.07)	6.52(−11.97, 27.87)	0.62(0.55–0.69)
South Asia	12481.18(8119.23–16643.79)	1.51(0.98, 1.98)	27079.60(22306.54–37097.27)	1.63(1.35, 2.24)	116.96(65.58–216.51)	8.49(−13.18, 57.74)	0.67(0.55–0.78)
Southeast Asia	6080.67(4373.36–7517.06)	1.82(1.33, 2.27)	14216.24(10393.57–17093.55)	2.08(1.54, 2.50)	133.79(82.78–181.19)	14.67(−6.78, 38.76)	1.46(1.39–1.53)
Southern Latin America	1242.61(1130.24–1356.37)	2.63(2.39, 2.87)	2306.44(2148.53–2456.24)	2.83(2.63, 3.02)	85.61(65.46–108.76)	7.61(−4.22, 20.72)	1.52(1.22–1.82)
Southern Sub-Saharan Africa	497.83(382.75–634.31)	1.46(1.10, 1.83)	1231.00(900.91–1461.89)	1.88(1.36, 2.22)	147.28(108.57–190.71)	29.20(11.19, 51.96)	1.61(1.51–1.70)
Tropical Latin America	4216.04(4007.25–4469.17)	3.69(3.51, 3.94)	12102.13(11477.48–12611.50)	4.80(4.55, 5.01)	187.05(167.95–205.51)	29.99(21.27, 38.13)	2.23(2.05–2.42)
Western Europe	21670.10(21088.96–22078.89)	4.33(4.24, 4.41)	33436.45(31382.03–34904.92)	4.34(4.14, 4.50)	54.30(47.48–59.88)	0.16(−3.08, 3.17)	1.02(0.95–1.09)
Western Sub-Saharan Africa	657.05(451.21–868.78)	0.39(0.28, 0.49)	2112.74(1104.12–2719.17)	0.56(0.31, 0.70)	221.55(95.46–303.14)	43.94(−6.98, 80.79)	1.02(0.90–1.14)
DALYs							
Global	5958480.98(4871421.75–6901954.98)	119.88(99.23, 137.57)	8912595.21(7612510.59–10356061.06)	107.91(91.74, 125.59)	49.58(28.22–69.92)	−9.99(−22.02, 1.24)	0.01(−0.03–0.04)
High SDI	1312839.95(1274152.52–1335629.44)	138.42(134.16, 140.88)	1781113.44(1708716.47–1852580.65)	121.28(117.00, 126.53)	35.67(31.18–40.13)	−12.39(−15.25, −9.29)	0.28(0.22–0.34)
High-middle SDI	1714311.63(1420345.98–1935786.74)	165.43(136.86, 186.78)	2227121.58(1905891.99–2570920.03)	139.39(119.25, 161.10)	29.91(11.42–50.08)	−15.74(−28.20, −2.39)	0.11(0.06–0.15)
Middle SDI	1947466.49(1457386.93–2303973.97)	123.37(93.26, 147.67)	2877442.75(2335479.14–3497678.72)	111.02(89.88, 134.25)	99.38(47.04–142.94)	−10.01(−25.64, 7.81)	0.07(0.02–0.12)
Low-middle SDI	739174.11(539761.71–1025046.66)	68.09(50.04, 94.17)	1473737.84(1172367.22–1835598.25)	81.83(65.23, 102.18)	29.91(11.42–50.08)	20.18(−7.38, 46.54)	0.68(0.65–0.71)
Low SDI	237683.48(149532.89–396649.95)	49.14(30.84, 76.19)	543980.76(373053.95–698811.71)	54.23(37.56, 69.66)	128.87(54.94–193.08)	10.35(−17.94, 34.28)	0.13(0.05–0.22)
Andean Latin America	38021.67(30031.16–53410.23)	106.44(85.40, 147.72)	88266.79(70226.70–109895.64)	137.13(109.15, 170.75)	132.15(59.96–209.95)	28.84(−7.96, 70.17)	1.11(0.81–1.41)
Australasia	37275.13(36193.85–38458.05)	173.89(168.88, 179.23)	56636.99(52907.51–60432.57)	142.13(133.15, 151.17)	51.94(41.99–63.60)	−18.27(−23.46, −12.46)	−0.03(−0.12–0.07)
Caribbean	32978.23(29026.70–46110.17)	100.78(90.24, 136.18)	59371.55(50989.35–71991.86)	120.40(102.12, 149.12)	80.03(47.32–107.06)	19.47(2.24, 36.13)	1.28(1.18–1.37)
Central Asia	86683.17(73173.83–100754.20)	131.46(110.10, 151.07)	176070.09(153099.39–202565.12)	182.25(158.84, 209.36)	103.12(65.82–150.51)	38.64(13.78, 72.09)	1.40(1.28–1.53)
Central Europe	278997.75(266528.38–297734.19)	208.80(199.43, 223.39)	303122.34(276834.27–330259.46)	186.47(169.76, 203.97)	8.65(−0.52–16.47)	−10.69(−18.88, −3.90)	0.64(0.49–0.80)
Central Latin America	121506.43(117568.84–126439.92)	81.28(79.06, 83.83)	231645.21(206113.30–261520.41)	90.87(80.70, 102.84)	90.64(68.89–114.63)	11.81(−1.10, 25.95)	0.58(0.40–0.76)
Central Sub-Saharan Africa	14013.59(9723.60–21831.96)	30.52(22.42, 41.39)	37964.95(25872.49–50473.48)	35.97(23.92, 49.18)	170.92(66.43–281.66)	17.87(−20.80, 56.64)	0.46(0.30–0.62)
East Asia	1925699.48(1364551.72–2345359.82)	171.93(122.56, 210.22)	2304510.40(1764774.92–2942851.53)	132.93(102.06, 169.28)	19.67(−7.36–55.14)	−22.68(−39.75, −0.47)	−0.20(−0.30--0.10)
Eastern Europe	341279.67(319141.85–359418.66)	143.55(135.10, 150.63)	412932.38(381399.02–448020.37)	156.28(144.98, 168.52)	21.00(8.14–37.02)	8.87(−2.06, 21.87)	0.78(0.68–0.87)
Eastern Sub-Saharan Africa	100937.55(67628.63–157894.93)	51.55(34.20, 73.61)	237287.92(172510.77–314192.70)	60.09(42.00, 77.85)	135.08(47.86–214.12)	16.56(−21.31, 45.18)	0.35(0.25–0.45)
High-income Asia Pacific	99317.00(83223.02–105742.98)	57.29(47.96, 61.29)	143487.10(124838.38–154975.11)	62.99(54.86, 67.63)	44.47(31.57–59.06)	9.94(−0.08, 21.33)	0.99(0.71–1.27)
High-income North America	480948.45(471488.06–488299.63)	158.96(156.25, 161.21)	665532.05(637599.28–683258.97)	134.63(130.28, 138.30)	38.38(34.61–41.60)	−15.30(−17.17, −13.41)	0.21(0.11–0.31)
North Africa and Middle East	422026.21(315098.94–603640.57)	146.05(111.15, 206.33)	879767.44(652323.34–1063847.60)	153.92(114.39, 185.97)	108.46(38.44–149.82)	5.39(−27.50, 28.02)	0.56(0.48–0.64)
Oceania	1464.50(718.39–2013.68)	25.70(12.67, 34.76)	3607.19(1909.84–4830.76)	28.13(14.76, 37.70)	146.31(99.83–198.89)	9.45(−9.36, 31.65)	0.48(0.42–0.53)
South Asia	656446.80(417584.73–895866.32)	64.14(41.51, 86.08)	1183609.95(959566.63–1598985.95)	67.11(54.35, 90.91)	80.31(30.84–159.58)	4.63(−20.17, 50.63)	0.10(0.00–0.20)
Southeast Asia	292573.11(204724.84–369849.11)	71.53(51.17, 88.97)	555833.62(402954.99–665412.45)	78.49(57.05, 93.86)	89.98(40.59–129.59)	9.73(−15.48, 31.40)	0.77(0.70–0.84)
Southern Latin America	46203.59(41942.79–50382.94)	95.43(86.64, 104.16)	74319.97(69347.98–79251.71)	100.03(93.34, 106.54)	60.85(44.21–80.42)	4.82(−6.18, 17.72)	1.00(0.72–1.28)
Southern Sub-Saharan Africa	22630.70(17664.39–29443.79)	53.42(41.19, 69.03)	50626.88(37684.08–61397.05)	69.14(51.20, 83.14)	123.71(84.77–163.82)	29.42(8.81, 52.86)	1.30(1.18–1.42)
Tropical Latin America	195772.86(184388.13–206372.25)	145.58(137.88, 153.40)	412631.87(394768.83–428785.20)	168.28(160.46, 175.42)	110.77(98.11–125.42)	15.60(8.94, 23.62)	1.22(1.00–1.43)
Western Europe	721095.49(708900.45–731625.66)	164.09(161.79, 166.30)	905401.01(868843.16–936528.66)	146.61(142.42, 151.33)	25.56(21.71–29.18)	−10.65(−13.25, −8.18)	0.35(0.29–0.42)
Western Sub-Saharan Africa	42609.60(28163.36–58275.44)	19.27(13.32, 24.95)	129969.51(66336.87–169011.75)	26.09(13.68, 33.62)	205.02(79.39–290.56)	35.42(−17.05, 69.92)	0.89(0.76–1.03)

### Regional level

The burden of Brain and CNS cancer varies significantly by region and is closely linked to SDI levels. From 1990 to 2021, the ASPR and ASIR increased progressively from the low SDI regions to the high SDI regions. In 2021, the high SDI regions had the highest prevalence, reaching 346,210.68 cases (95% UI, 325,277.68–361,913.90), with an ASPR of 26.10 (95% UI, 24.84–27.19) per 100,000 population and an EAPC of 1.72% (95% CI, 1.62–1.83%). In contrast, the low SDI regions had 26,499.66 cases (95% UI, 18,495.16–33,547.10), an ASPR of 2.53 (95% UI, 1.76–3.24) per 100,000, and an EAPC of 0.64% (95% CI, 0.51–0.78%). Similarly, the high SDI regions had the highest incidence, reaching 100,993.68 cases (95% UI, 94,963.67–105,628.62), with an ASIR of 6.38 (95% UI, 6.08–6.64) per 100,000 and an EAPC of 1.25% (95% CI, 1.19–1.31%), while the low SDI regions had the lowest incidence at 55,694.12 cases (95% UI, 54,173.05–56,847.27), with an ASIR of 1.43 (95% UI, 1.00–1.82) per 100,000 and an EAPC of 1.25% (95% CI, 1.19–1.31%). In contrast, the highest ASDR and age-standardized DALY rates were observed in high-middle SDI regions in 2021, with an ASDR of 3.94 (95% UI, 3.36–4.51) per 100,000 and an age-standardized DALY rate of 139.39 (95% UI, 119.25–161.10) per 100,000. However, compared to 1990, both the high SDI and high-middle SDI regions showed declines in ASDR and age-standardized DALY rates, and the age-standardized DALY rate also decreased in middle SDI regions. In low SDI regions, the ASDR was 1.33 (95% UI, 0.93–1.69) per 100,000, and the age-standardized DALY rate was 54.23 (95% UI, 37.56–69.66) per 100,000 (Table 1; Figure 2). Among the five SDI regions in 2021, the ASPR was higher in males than in females in most regions, and the ASIR, ASDR, and age-standardized DALY rates were all higher in males than in females (Supplementary Figure 9; Supplementary Table S1).

**Figure 3:**
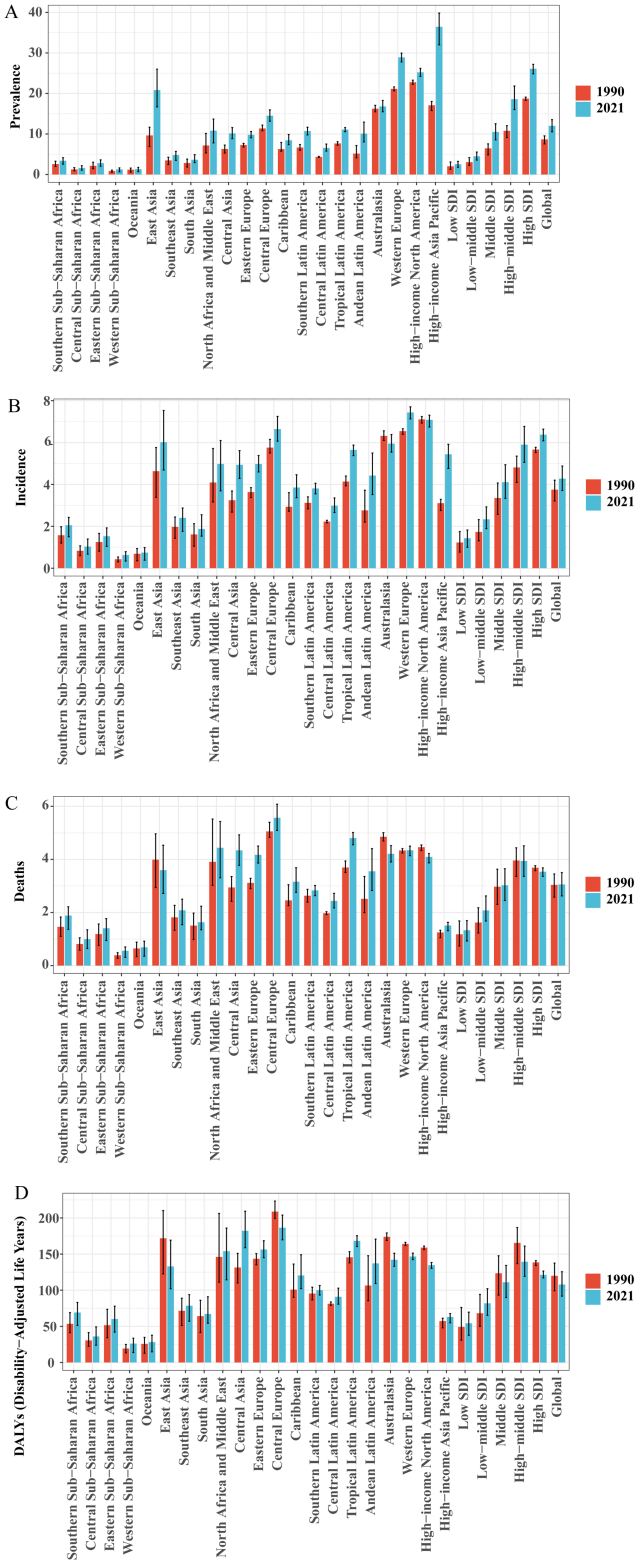
Comparison of ASPR, ASIR, ASDR, and DALY rates of Brain and CNS cancer across global regions in 1990 and 2021. **(A)** ASPR; **(B)** ASIR; **(C)** ASDR; **(D)** DALYs rate.

The 21 GBD regions are made up of countries and territories that are geographically close and epidemiologically similar. Among the 21 regions, our findings indicate that the High-income Asia Pacific region bears the highest age-standardized prevalence rate (ASPR) of Brain and CNS cancer globally. Specifically, the ASPR reached 36.44 (95% UI, 32.00–39.83) per 100,000 in 2021, an increase of 113.53% compared to 17.07 (95% UI, 15.78–18.04) per 100,000 in 1990. This is followed by Western Europe (28.90 [95% UI, 27.78–29.95] per 100,000) and High-income North America (25.21 [95% UI, 24.20–26.19] per 100,000), ranking second and third, respectively. In our analysis, the age-standardized incidence rate (ASIR) in Europe is noteworthy, as Western Europe and Central Europe rank first and third globally, with ASIRs of 7.44 (95% UI, 7.13–7.71) and 6.64 (95% UI, 6.07–7.25) per 100,000, respectively, while High-income North America ranks second with an ASIR of 7.08 (95% UI, 6.75–7.31) per 100,000. Among all regions, High-income Asia Pacific showed the fastest ASIR growth, increasing from 3.11 (95% UI, 2.76–3.29) per 100,000 in 1990 to 5.44 (95% UI, 4.76–5.92) per 100,000 in 2021—a growth rate of 75.13%—with an estimated annual percent change (EAPC) of 3.46% (95% CI, 3.14–3.78%). East Asia, Australasia, and Tropical Latin America also demonstrated relatively high ASIRs. Central Europe exhibited the highest age-standardized mortality rate (ASDR) at 5.57 (95% UI, 5.09–6.08) per 100,000, followed by Tropical Latin America at 4.80 (95% UI, 4.55–5.01) per 100,000, and North Africa and Middle East at 4.44 (95% UI, 3.31–5.43) per 100,000. Between 1990 and 2021, a decline in ASDR was observed in High-income North America, East Asia, and Australasia. Similar to ASDR, the highest age-standardized DALY rate was recorded in Central Europe (186.47 [95% UI, 169.76–203.97] per 100,000), followed by Central Asia (182.25 [95% UI, 158.84–209.36] per 100,000) and Tropical Latin America (168.28 [95% UI, 160.46–175.42] per 100,000). Compared with 1990, some regions—such as Western Europe, High-income North America, East Asia, Central Europe, and Australasia—showed a downward trend in the age-standardized DALY rate. This decline may be attributed to advances in comprehensive treatment techniques, standardized diagnostic and treatment procedures, and improved early detection and intervention, which collectively reduce mortality risk and decrease DALYs associated with brain tumors. At the same time, population aging and improved case detection could lead to increases in incidence and prevalence. Likewise, among the 21 regions, most show higher ASPR in males than in females. However, ASIR is higher in females than in males only in Eastern Sub-Saharan Africa and Western Sub-Saharan Africa. Apart from Western Sub-Saharan Africa, the age-standardized DALY rate is higher in males in all other regions (Figure 3; Supplementary Figure 10; Supplementary Table S2).

### National level

Across all countries worldwide, there is considerable variation in the ASPR, ASIR, ASDR, and age-standardized DALY rates of Brain and CNS cancers. In 2021, the ASPR ranged from approximately 0.30 to 98.34 per 100,000. Norway had the highest ASPR at 98.34 (95% UI, 91.47–106.14) per 100,000, followed by Denmark (88.84 [95% UI, 80.72–97.45]) and Monaco (71.71 [95% UI, 49.22–95.75]). Notably, all three countries are in Europe, aligning with our previous region-level findings, which identified Europe as having the heaviest ASPR burden globally. Similar trends were observed for ASIR, with Norway (14.96 [95% UI, 13.93–16.07] per 100,000), Denmark (14.16 [95% UI, 12.90–15.42]), and Monaco (13.91 [95% UI, 9.62–18.37]) as the top three, whereas The Gambia had the lowest ASIR at 0.14 (95% UI, 0.09–0.19) per 100,000. This further confirms that the highest incidence rates are clustered in European nations.

Regarding ASDR and age-standardized DALY rates, the top three countries in 2021 were Montenegro, North Macedonia, and Bulgaria. Montenegro had the highest ASDR at 7.77 (95% UI, 6.11–10.25) per 100,000, with an age-standardized DALY rate of 276.39 (95% UI, 211.29–363.85) per 100,000. North Macedonia followed with an ASDR of 7.55 (95% UI, 5.14–9.99) and a DALY rate of 263.84 (95% UI, 187.40–364.89) per 100,000. Bulgaria ranked third, with an ASDR of 7.47 (95% UI, 6.21–8.94) per 100,000 and an age-standardized DALY rate of 261.72 (95% UI, 215.55–311.68) per 100,000 (Figure 3; Supplementary Table S3).

**Figure 4:**
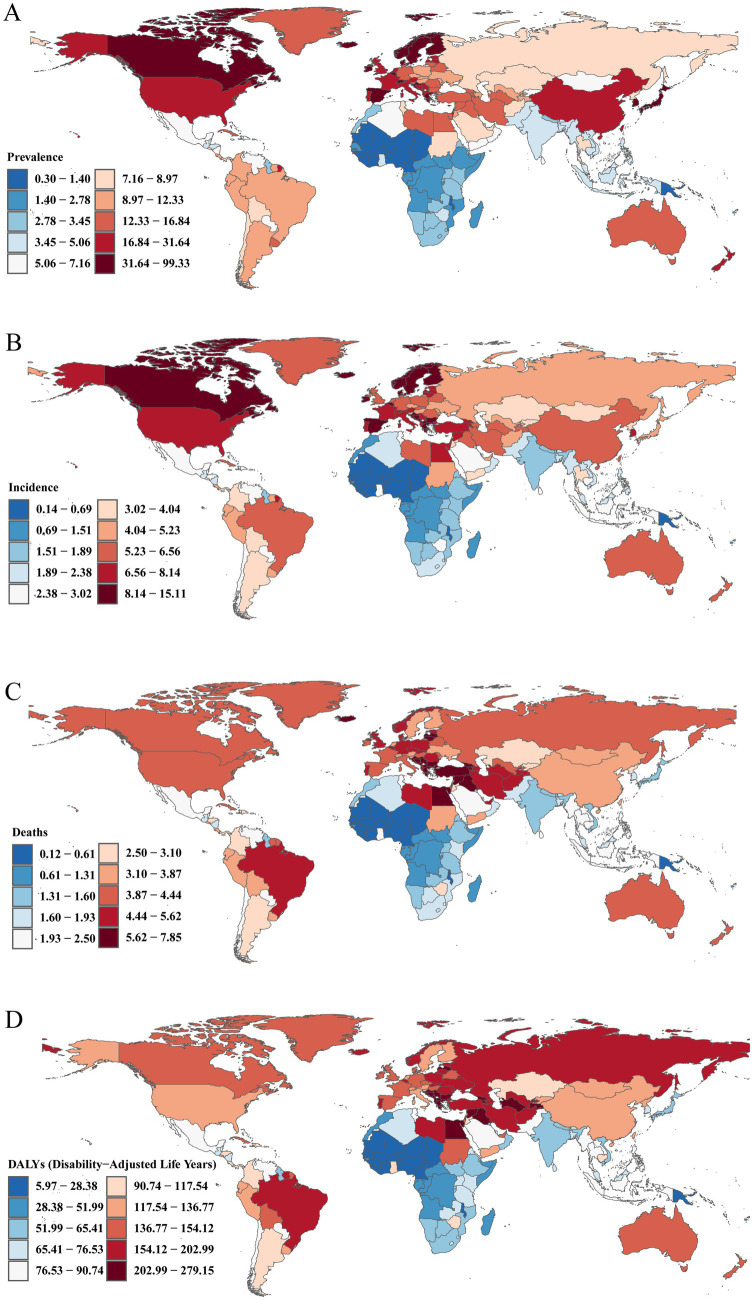
Maps depicting the burden of Brain and CNS cancer across 204 countries and territories. **(A)** ASPR; **(B)** ASIR; **(C)** ASDR; **(D)** Age-standardized DALY rate.

From 1990 to 2021, the Republic of Ecuador showed the most pronounced increase in ASPR, with an age-standardized EAPC of 7.69% (95% CI, 5.58–9.85%), followed by Turkmenistan (7.51%). In terms of ASIR, Turkmenistan had the highest EAPC at 7.44% (95% CI, 6.31–8.58%), closely followed by the Republic of Ecuador (7.38%). The same pattern held for ASDR and age-standardized DALY rates, where Turkmenistan and the Republic of Ecuador also occupied the top two positions. Turkmenistan’s EAPCs were 7.30% (95% CI, 6.14–8.48%) and 6.95% (95% CI, 5.87–8.04%), respectively, while those of the Republic of Ecuador were 7.17% (95% CI, 5.03–9.34%) and 6.59% (95% CI, 4.49–8.73%). Notably, Georgia ranked third for all four indicators. Among all countries, Greenland exhibited the most substantial decrease in age-standardized EAPCs for ASPR, ASIR, ASDR, and DALY rates, with values of −0.71% (95% CI, −1.02% to −0.40%), −1.29% (95% CI, −1.48% to −1.10%), −1.63% (95% CI, −1.79% to −1.47%), and −1.83% (95% CI, −2.02% to −1.64%), respectively (Supplementary Table S4; Supplementary Figure S11). In terms of overall percentage changes in ASPR, ASIR, ASDR, and age-standardized DALY rates from 1990 to 2021, the Republic of Ecuador again showed the fastest growth at 902.14, 853.12, 793.01, and 644.27%, respectively, whereas Greenland showed the largest declines at −25.70, −35.45%, −41.29%, and −44.67%, respectively (Supplementary Table S5; Figure 4).

### Age and sex patterns

**Figure 5:**
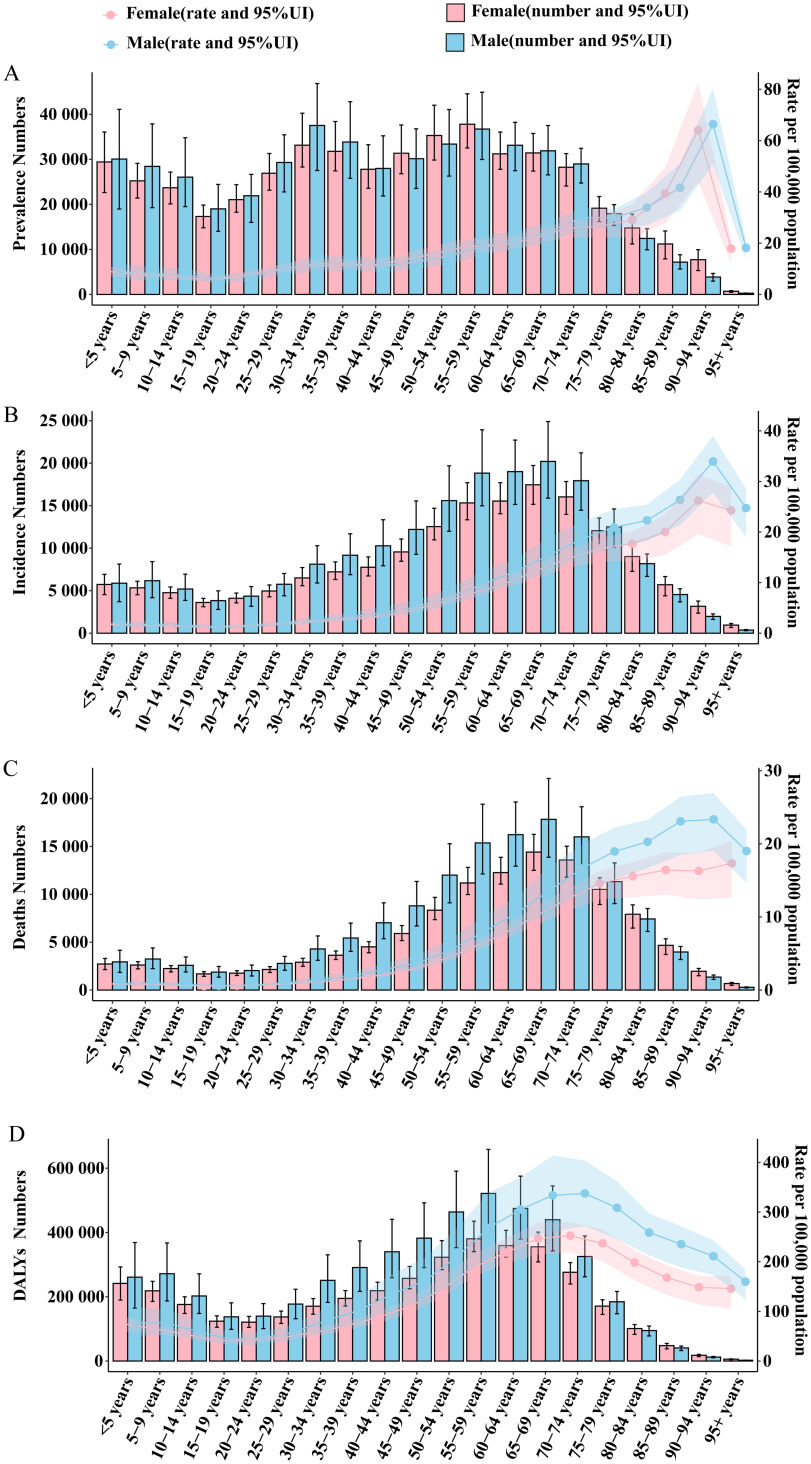
Global trends in the number and rate of Brain and CNS cancer prevalence, incidence, mortality, and DALYs by age and sex in 2021. **(A)** Number of prevalent cases and ASPR; **(B)** Number of incident cases and ASIR; **(C)** Number of deaths and ASDR; **(D)** Number of DALYs and ASR.

In 2021, the global number of male and female Brain and CNS cancer cases in children and adults younger than 75 remained relatively high, showing a slight decrease at ages 15–19 and a more pronounced decline after age 75. By contrast, the prevalence rate increased with age, peaking in both males and females at 90–94 years. Overall, there was no significant difference between the sexes in terms of absolute numbers and ASPR (Figure 5). In the High SDI region, both the number of cases and ASPR increased with age, with the number of cases peaking at 70–74 years and then declining, while the prevalence rate peaked at 90–94 years. Moreover, up to age 80, the number of male cases exceeded that of female cases; after age 80, female cases surpassed male cases. The ASPR was higher in males than in females, and the difference widened with age. In the High-Middle SDI region, the number of cases was higher between ages 30 and 70. After age 40, the number of cases in females exceeded that in males. Before age 20–24, the ASPR declined slowly and was similar between males and females. After reaching adulthood, the ASPR rose gradually, although without large fluctuations, and in most age groups, females had a higher ASPR than males. In the Middle SDI region, the number of cases increased markedly before age 15, then remained high between ages 30 and 60. The ASPR also rose significantly in children; it declined before age 20 and then climbed again with some fluctuations. Between ages 45 and 60, females had a notably higher ASPR than males, with little difference outside this range. In Low-Middle SDI and Low SDI regions, the number of pediatric cases was much higher than that of adults and older adults. Incidence in these regions decreased with age during childhood, rose again in adulthood, and then declined in older age. In the Low-Middle SDI region, the number of male cases and ASPR exceeded those of females for most age groups. By contrast, in Low SDI regions, the difference in ASPR between males and females was minimal (Supplementary Figure 12).

Globally and in High SDI regions, both the number of new cases and ASIR of Brain and CNS cancer exhibited a broadly similar pattern, rising with age. Globally, the number of new cases peaked at ages 65–69, whereas in High SDI regions, it peaked at 70–74, then declined with advancing age. Up to age 80, the number of male cases remained higher than that of female cases in both global and High SDI populations. The ASIR also increased with age, peaking at 90–94 years worldwide and in High SDI regions, and the difference in incidence between males and females grew with age, favoring males. High-Middle SDI and Middle SDI regions followed similar overall patterns, although the peak number of cases shifted one or two age groups earlier than in High SDI regions. Overall, the number of cases remained higher in males, particularly in adulthood. In Middle SDI regions, the number of pediatric cases gradually increased, and the ASIR rose with age. The gap between male and female ASIRs widened progressively, with males showing higher rates. Like the trends in prevalence, pediatric incidence in Low-Middle SDI and Low SDI regions increased substantially, especially in Low SDI regions, where the number of pediatric cases was much higher than in other age groups. In Low-Middle SDI regions, the incidence in males and the ASIR were still higher than in females, and the male–female difference expanded in older age groups. By contrast, in Low SDI regions, the difference between males and females was negligible (Figure 5; Supplementary Figure 13). Likewise, mortality patterns generally mirrored those of prevalence and incidence. In the global, High SDI, High-Middle SDI, and Middle SDI regions, overall deaths remained higher among males than females, with female mortality surpassing male mortality only in the oldest age groups. Moving from High SDI to Middle SDI regions, as the SDI index decreased, the peak age range for the number of deaths shifted one or two age groups earlier. The ASDR rose with age and reached its maximum at 90–94 years, with the gap between male and female mortality widening with age, favoring males. Again, consistent with the patterns in prevalence and incidence, child mortality climbed markedly in Low-Middle SDI and Low SDI regions, particularly in Low SDI regions where the number of deaths among children was much higher than at other ages. In Low-Middle SDI regions, the ASDR increased steadily with age, and both the number of male deaths and the ASDR remained higher in males. Low SDI regions also showed a relatively high ASDR, although the difference between males and females was small. Across all regions, the DALY patterns by age and sex closely followed previous trends, but at the global level, the DALY count and rate among children under 10–14 years were notably high. In the Middle SDI region, DALYs among children were already higher than in High-Middle and High SDI regions (Figure 5; Supplementary Figures 14, 15).

### Decomposition analysis

**Figure 6:**
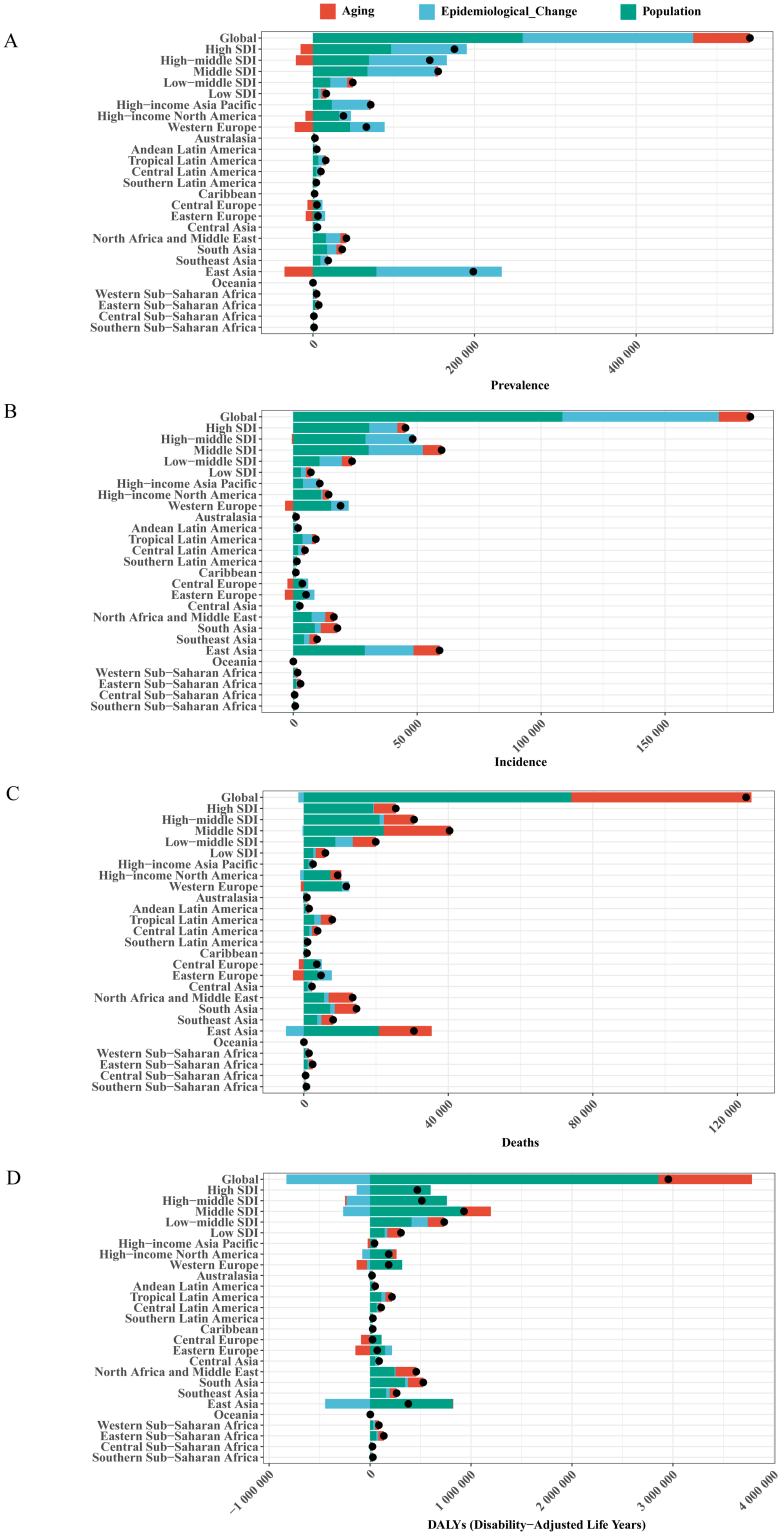
Changes in Brain and CNS cancer burden from 1990 to 2019 attributable to population growth, aging, and epidemiological changes. The black dot indicates the total change contributed by all factors. **(A)** prevalence; **(B)** incidence; **(C)** deaths; **(D)** DALYs. The black dot represents the total change contributed by all three factors. Positive values indicate an increase in Brain and CNS cancer attributable to that factor, while negative values indicate a decrease.

From 1990 to 2021, the global numbers of prevalent cases, incident cases, deaths, and DALYs related to Brain and CNS cancer all showed a significant increase. Regarding prevalence, among the five SDI regions, the High SDI region experienced the largest increase, while among the 21 GBD regions, East Asia had the greatest increase. Overall, aging, population growth, and epidemiological changes contributed 12.99, 48.00, and 39.01%, respectively, to the global increase in prevalence. Central Europe was most influenced by population growth (137.45%), while aging exerted the largest negative impact (−137.61%). Eastern Europe was most affected by epidemiological changes (114.55%). For incidence, the Middle SDI region had the greatest increase among the five SDI groups, whereas East Asia again led among the 21 regions. Aging, population growth, and epidemiological changes accounted for 6.88, 58.89, and 34.24% of the global rise in incidence, respectively. The Low SDI region saw the most significant contribution from aging (28.97%) among the five SDI groups, and Oceania (47.61%) among the 21 regions. Population growth had the largest impact in the High SDI region (67.67%) and Central Europe (104.62%), while epidemiological changes contributed most in the High-middle SDI region (40.65%) and Eastern Europe (77.52%). Concerning mortality, aging and population growth contributed 40.72 and 60.49%, respectively, to the global increase in deaths. Among the five SDI regions, the most substantial contribution from aging occurred in the High SDI region (75.36%), while the largest impact of population growth was observed in the Low-middle SDI region (24.22%). As for DALYs, aging and population growth contributed 31.36 and 96.68%, respectively, to the global increase. Among the five SDI groups, aging had the highest contribution in the High-middle SDI region (148.37%), whereas population growth made the most significant impact in the Low-middle SDI region (21.80%). Epidemiological changes had a negative effect on the global number of deaths and DALYs (−1.22% and −28.04%, respectively). Middle SDI made the greatest negative contribution to deaths (−0.82%), while High-middle SDI showed the largest negative contribution for DALYs (−45.32%). Demographic and epidemiological factors influenced the prevalence, incidence, mortality, and DALYs of Brain and CNS cancer differently across countries and regions (Figure 6; Supplementary Table S6).

### Cross-national inequality analysis

**Figure 7:**
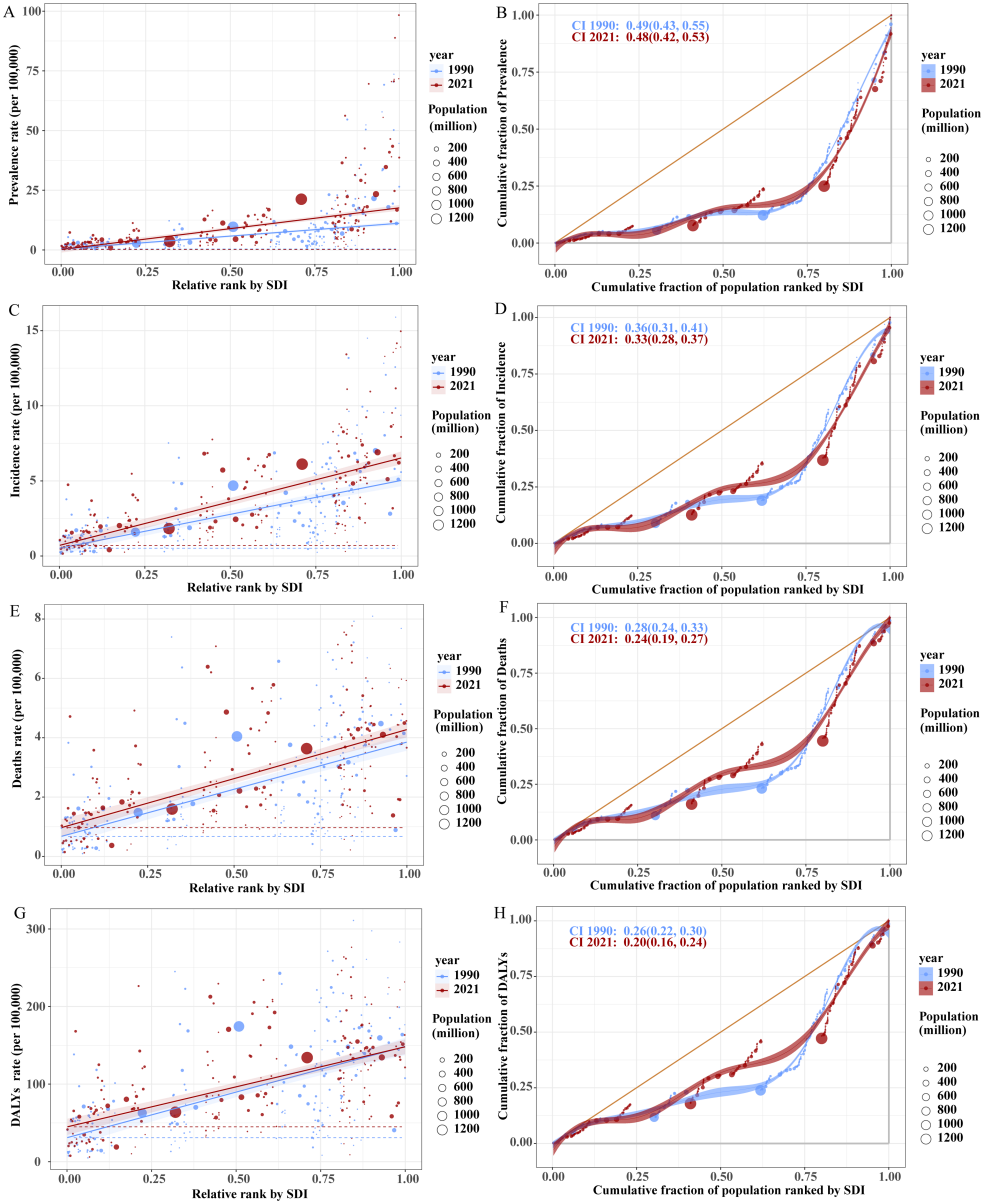
Health inequality regression curves (left) and concentration curves (right) for the global age-standardized prevalence (ASPR; **A,B**), incidence (ASIR; **C,D**), mortality (ASDR; **E,F**), and DALY rates **(G,H)** of Brain and CNS cancer from 1990 to 2021.

From 1990 to 2021, the prevalence burden of Brain and CNS cancer gradually increased, with a pronounced rise in high-SDI countries and a moderate but noticeable increase in low-SDI countries. In 1990, the slope index of inequality was 10.78, which expanded to 17.44 by 2021. This indicates that, compared with the country/region having the lowest SDI in 1990, the country/region with the highest SDI had 17.44 more prevalent cases per 100,000 population in 2021. However, the prevalence concentration index in 2021 (0.49) was slightly lower than that in 1990 (0.52), suggesting that the burden in low-SDI countries has grown but remains largely concentrated in high-SDI countries. A similar pattern was observed for incidence: the slope index of inequality increased from 4.52 in 1990 to 5.82 in 2021, whereas the concentration index decreased marginally from 0.35 in 1990 to 0.32 in 2021. Mortality rates likewise rose over time, remaining higher in high-SDI regions than in low-SDI regions, with a slight decline in the mortality concentration index. In contrast, both the slope index of inequality and the concentration index for DALYs decreased, indicating that by 2021, overall regional inequalities in DALY burden had narrowed worldwide (Figure 7).

### Future forecasts of global burden of disease in brain and CNS cancer

**Figure 8:**
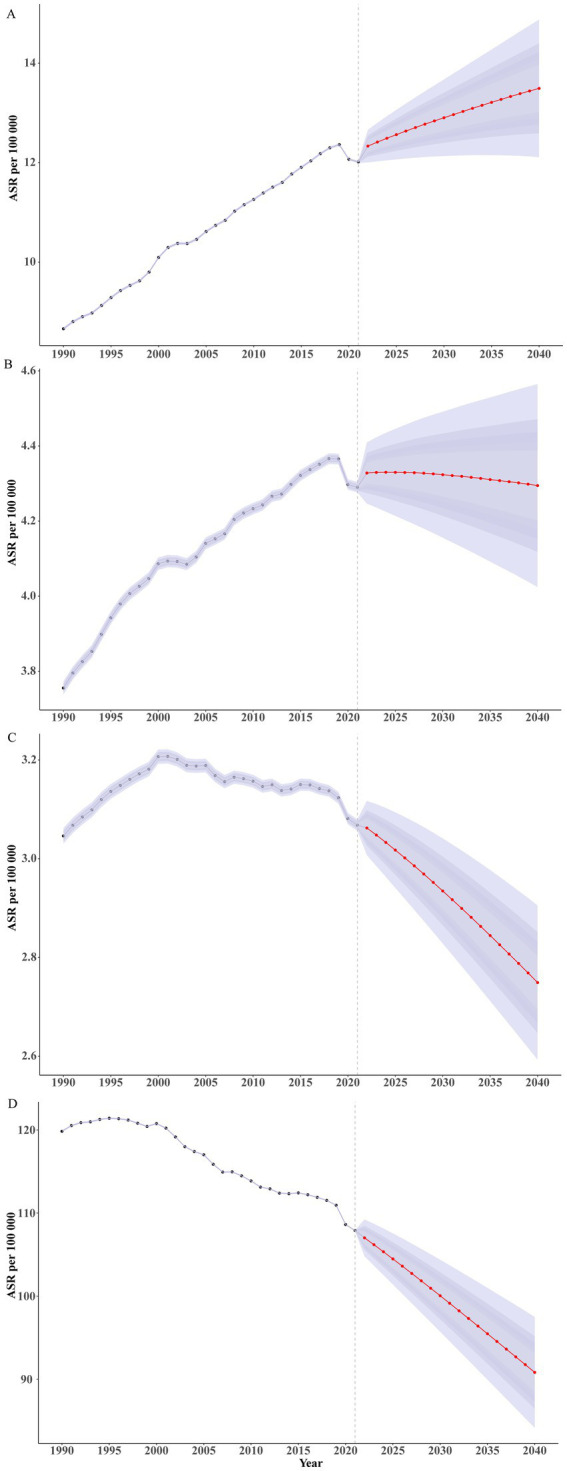
Projections for the burden of Brain and CNS cancer by 2040. **(A)** ASPR; **(B)** ASIR; **(C)** ASDR; **(D)** Age-standardized DALY rate.

From 2021 to 2040, the global burden of Brain and CNS cancer is projected to remain relatively stable. The global ASPR for both sexes is expected to continue its gradual increase, rising from approximately 12.01 per 100,000 in 2021 to about 13.49 per 100,000 in 2040. The ASPR for females is projected to increase from around 11.77 per 100,000 to about 13.55 per 100,000, whereas for males it is projected to rise from approximately 12.29 per 100,000 to 13.52 per 100,000. The increase in females is slightly higher than that in males. The global ASIR for Brain and CNS cancer is predicted to change little overall. Male ASIR may decrease slightly, from about 4.74 per 100,000 in 2021 to around 4.69 per 100,000 in 2040, whereas female ASIR is expected to increase marginally from about 3.89 per 100,000 in 2021 to 3.94 per 100,000 in 2040, with minimal change in the gap between sexes. By contrast, the total ASDR for Brain and CNS cancer is projected to decrease from approximately 3.07 per 100,000 in 2021 to about 2.75 per 100,000 in 2040, with an ASDR of around 3.19 per 100,000 in males and 2.35 per 100,000 in females by 2040. The age-standardized DALY rate for Brain and CNS cancer is also anticipated to decline slightly, from about 107.89 per 100,000 in 2021 to approximately 90.83 per 100,000 in 2040. In 2040, the male age-standardized DALY rate (approximately 102.55 per 100,000) is expected to exceed that of females (about 79.83 per 100,000; Figure 8; Supplementary Table S7). By age group, the ASPR in children is projected to decline slowly through 2040, whereas it will continue to rise in adults and older adults (Supplementary Table S8; Supplementary Figure 16). The ASIR is expected to decrease before age 35, increase between ages 40 and 60, and remain relatively stable in older adults (Supplementary Table S9; Supplementary Figure 17). With future improvements in medical technology and the availability of new treatments, the ASDR (Supplementary Table S10; Supplementary Figure S18) and age-standardized DALY rate (Supplementary Table S11; Supplementary Figure 19) are expected to continue declining before age 80 and remain stable at very advanced ages.

## Discussion

Although Brain and CNS cancers are relatively rare, they have high lethality and disability rates, posing serious threats to physical and mental health. Our comprehensive analysis of the global burden of Brain and CNS cancer from 1990 to 2021 reveals a complex situation characterized by variations and trends across different regions and levels of Sociodemographic Index (SDI). Globally, the ASPR and ASIR for Brain and CNS cancer have shown an upward trend, only slightly declining around 2018 but overall remaining relatively high. The ASDR initially increased and then decreased, and, compared with 1990, the ASDR in 2021 was only slightly higher. Meanwhile, the age-standardized DALY rate declined from 1990 to 2021, with relative percentage changes of 38.66, 16.09, 0.60%, and −9.99% for ASPR, ASIR, ASDR, and the DALY rate, respectively. The global increases in ASPR and ASIR may be attributed to advancements in medical facilities and technologies, the emergence of new diagnostic equipment and methods, more refined disease classification, and clearer diagnostic criteria—all contributing to improved detection and screening of Brain and CNS cancers (18). In addition, population growth and an aging society (19), along with industrial development (e.g., ionizing radiation exposure, pesticides, oil products, rubber or vinyl chloride, mobile phone use) and environmental changes (e.g., contaminated drinking water, lead exposure) are also driving incidence rates higher (20). At the regional level, the High SDI regions exhibit the highest ASPR and ASIR, whereas the highest ASDR and age-standardized DALY rate are found in High-middle SDI regions, and the lowest values in Low SDI regions. These findings underscore persistent socioeconomic disparities in the global burden of Brain and CNS cancer. High SDI regions generally benefit from more advanced healthcare facilities and technologies, along with improved clinical pathological and molecular diagnostic tools—for instance, 64-slice or even more powerful multi-detector CT scans, high-field-strength MRI, functional MRI (fMRI), and diffusion-weighted imaging (DWI)—which can provide clearer brain structure imaging and more accurate identification of tumor location, size, boundaries, and invasiveness. Further, brain biopsy, immunohistochemistry, NGS sequencing, and PCR testing can facilitate more precise prognostic evaluation of Brain and CNS cancers (21, 22). Additionally, greater awareness of early detection and enhanced screening, more comprehensive cancer registration and follow-up systems, and broader access to healthcare services can all contribute to the higher ASIR and ASPR observed in these regions (23). In High-middle SDI regions, the highest ASDR and DALY rate may reflect the limited availability or effectiveness of treatment: while diagnosis rates are relatively high, treatment outcomes may be suboptimal, leading to poorer quality of life and survival, thus elevating ASDR and DALY rates. In Low SDI regions, by contrast, the scarcity of healthcare resources and cancer screening technologies may result in many cases remaining undiagnosed; coupled with the poor prognosis and short survival times of Brain and CNS cancers, these areas show the lowest observed rates (24, 25). Moreover, High SDI regions also have relatively high ASDR and DALY rates, suggesting that, despite significant advances in surgery, molecular targeting, and immunotherapies, survival outcomes for Brain and CNS cancers remain disappointing (10). Notably, the slight decline in ASPR and ASIR between 2018 and 2021 may be partially attributable to the COVID-19 pandemic. During this period, a substantial portion of healthcare personnel and medical resources was redirected toward treating and screening COVID-19 patients, causing delays or cancelations in imaging examinations, surgeries, and pathological diagnoses. Consequently, some Brain and CNS cancer cases that might have been detected and confirmed during this period were instead postponed or missed. Furthermore, public concerns about the risk of cross-infection led to fewer hospital visits, while public health authorities shifted their focus to pandemic control, potentially delaying updates and data integration for cancer registries and follow-up (26, 27).

At the national level, Norway, Denmark, and Monaco had the highest ASPR and ASIR, while Montenegro, North Macedonia, and Bulgaria showed the highest ASDR and age-standardized DALY rates, all of which are European countries. This finding is consistent with our previous analysis. Norway, Denmark, and Monaco are considered high or even very high SDI countries globally, whereas Montenegro, North Macedonia, and Bulgaria belong to high-middle SDI regions. Even within Europe, socioeconomic imbalances affect disease burden. With an aging population, residents in high SDI countries can afford and access comprehensive multidisciplinary treatment, including early screening, surgery, radiotherapy, chemotherapy, and rehabilitation follow-up. Meanwhile, in some less economically developed countries, patients often struggle to receive timely and standardized treatment, ultimately leading to steep increases in ASDR and age-standardized DALY rates (28). The most rapid increases in ASPR, ASIR, ASDR, and age-standardized DALY rates occurred in the Republic of Ecuador, Turkmenistan, and Georgia, which are categorized under high-middle SDI or middle SDI regions. This could be related to social progress, population growth, and the transition to or challenges of an aging society, as well as the environmental pollution and lifestyle changes (e.g., smoking, alcohol abuse, unhealthy diets, and obesity) that accompany urbanization and industrialization. These factors collectively alter the epidemiological profile, with a rising proportion of non-communicable diseases. These findings align with our cross-national inequality analysis, which indicates that, despite a minor reduction in disparities over the past 2 years, there remains a substantial overall gap between regions with higher SDI indices and those with lower SDI indices.

Globally and in most regions, the overall ASIR, ASDR, and age-standardized DALY rates of Brain and CNS cancer are higher in males than in females, and the absolute number of cases in males also exceeds that in females. These differences may be attributable to factors such as sex-specific metabolic and hormonal differences, variations in immune microenvironments, genetic and molecular biological distinctions, as well as environmental and lifestyle factors. For example, the rate of cellular and organismal metabolism and growth varies by sex, and research indicates that surgical samples from male glioblastoma (GBM) patients contain higher levels of amino acid metabolites (including glutamine), influencing a significantly higher glutamine uptake in male gliomas (29). Estrogen may have a protective effect on glioma patients, whereas androgen exposure could increase the risk of glioma (30). There are pronounced differences in immune function between males and females, and emerging evidence suggests that T cells play a pivotal role in driving GBM’s sex-specific differences. A higher frequency of progenitor-exhausted T cells has been observed in males, potentially modulating the response to anti-PD-1 therapy. These intrinsic differences might be linked to sex chromosomes, possibly through gene expression that escapes X-chromosome inactivation (XCI) or via microRNAs highly enriched on the X chromosome (31). Additionally, factors like the methylation status of O^6-methylguanine-DNA methyltransferase (MGMT) and the tumor suppressor gene TP53 may be critical in the sex-related malignant behaviors and prognosis of gliomas (30). Moreover, men often work in harsher conditions with greater exposure to pesticides, chemicals, and biological agents, and they may also engage in behaviors such as smoking and heavy drinking (although the direct impact on Brain and CNS cancer remains debated), all of which may contribute to these observed sex differences (32).

It is noteworthy that the burden of ASPR, ASIR, ASDR, and age-standardized DALYs among children (0–14 years) is more pronounced in regions with lower SDI values, particularly in the Low SDI areas. As the SDI increases, the age at which Brain and CNS cancer exerts the greatest burden also increases. Ionizing radiation poses a stronger carcinogenic risk for children due to their higher sensitivity to radiation, and limited public health education and health promotion in Low SDI regions exacerbate the situation. For instance, diagnostic radiotherapy administered to pregnant women has been linked to an increased risk of primary brain tumors. In addition, pediatric brain tumors are associated with structural birth defects: non-chromosomal structural birth defects (excluding trisomies and other chromosomal anomalies) are among the strongest and most consistent risk factors for childhood cancer, with about 7% of childhood brain tumors being attributable to such defects. Although established risk factors for childhood brain tumors remain largely limited to ionizing radiation exposure and certain cancer syndromes, growing evidence points to positive correlations with advanced parental age, birth defects, fetal growth markers, maternal dietary nutrition, residential pesticide exposure, and parental smoking. Many of these issues are potentially more common in Low SDI areas due to weaker socioeconomic development (33–37).

This study employs epidemiological models to analyze the global disease burden of Brain and CNS cancers, offering valuable insights for countries and healthcare professionals to formulate age- and sex-specific preventive measures, management policies, and diagnostic and treatment strategies. However, it also has several limitations. First, data loss is inevitable in statistics across different regions. In many low and middle-income countries, inadequate cancer registration leads to under reporting of cases, while limited health care resources—such as insufficient diagnostic equipment, uneven distribution of medical facilities, and shortages of trained personnel—result in misdiagnosis or missed diagnoses. The international community should therefore strengthen collaboration with these areas, support the development of diagnostic and data collection infrastructure, invest in workforce training and health education, and release regular regional data quality assessment reports to advance global health efforts. Second, because the data come from multiple countries, and the diagnosis and classification of Brain and CNS cancers are inherently complex, variations in statistical agencies and health authorities affect data sources and accuracy. We recommend wider adoption of the WHO’s latest CNS tumor classification and diagnostic standards, as well as the establishment of a data-sharing platforms to enhance the comparability and reliability of data. In addition, although our analysis covered global data, we did not provide a detailed explanation of the factors driving key temporal trends across age groups, regions, and countries. Future investigations should refine analyses by specific age brackets, geographic areas, and individual nations. The GBD methodology also relies on multiple assumptions and modeling techniques, which can introduce bias and uncertainty. More comprehensive research into the etiology and risk factors of brain and CNS cancers is needed, particularly large scale randomized controlled trials examining environmental, lifestyle, and genetic determinants. Clarifying regional differences in prevention, causation, and clinical management will facilitate the development of targeted interventions for brain and CNS cancers.

## Conclusion

In summary, although the ASDR and age-standardized DALY rates for Brain and CNS cancer have shown a declining trend, the absolute burden of the disease remains substantial, with marked variations across different regions, countries, and SDI levels. Children in low-income regions bear a heavy burden, while older adults in high-income regions are disproportionately affected. These findings underscore that strengthening healthcare systems and reducing socioeconomic disparities are critical steps toward mitigating the global burden of Brain and CNS cancers. Future research should prioritize identifying the most effective interventions and policies for Brain and CNS cancer prevention and management, with additional focus on genetic and sex-related differences.

## Data Availability

Publicly available datasets were analyzed in this study. GBD study 2021 data resources were available online from the Global Health Data Exchange (GHDx) query tool (http://ghdx.healthdata.org/gbd-results-tool). The results related to this study are presented in the Supplementary materials.

## Footnotes

^1^ https://vizhub.healthdata.org/gbd-results/
