# Supplementary material for: The global, regional, and national brain and central nervous system cancer burden and trends from 1990 to 2021: an analysis based on the Global Burden of Disease Study 2021
Source: Front Neurol. 2025 Jun 18;16:1574614. doi: 10.3389/fneur.2025.1574614 (PMC12213423; doi:10.3389/fneur.2025.1574614)
Supplement: Supplementary file 1 [file Data_Sheet_1.zip › Supplementary Data/Supplementary Fig. 10.pdf]

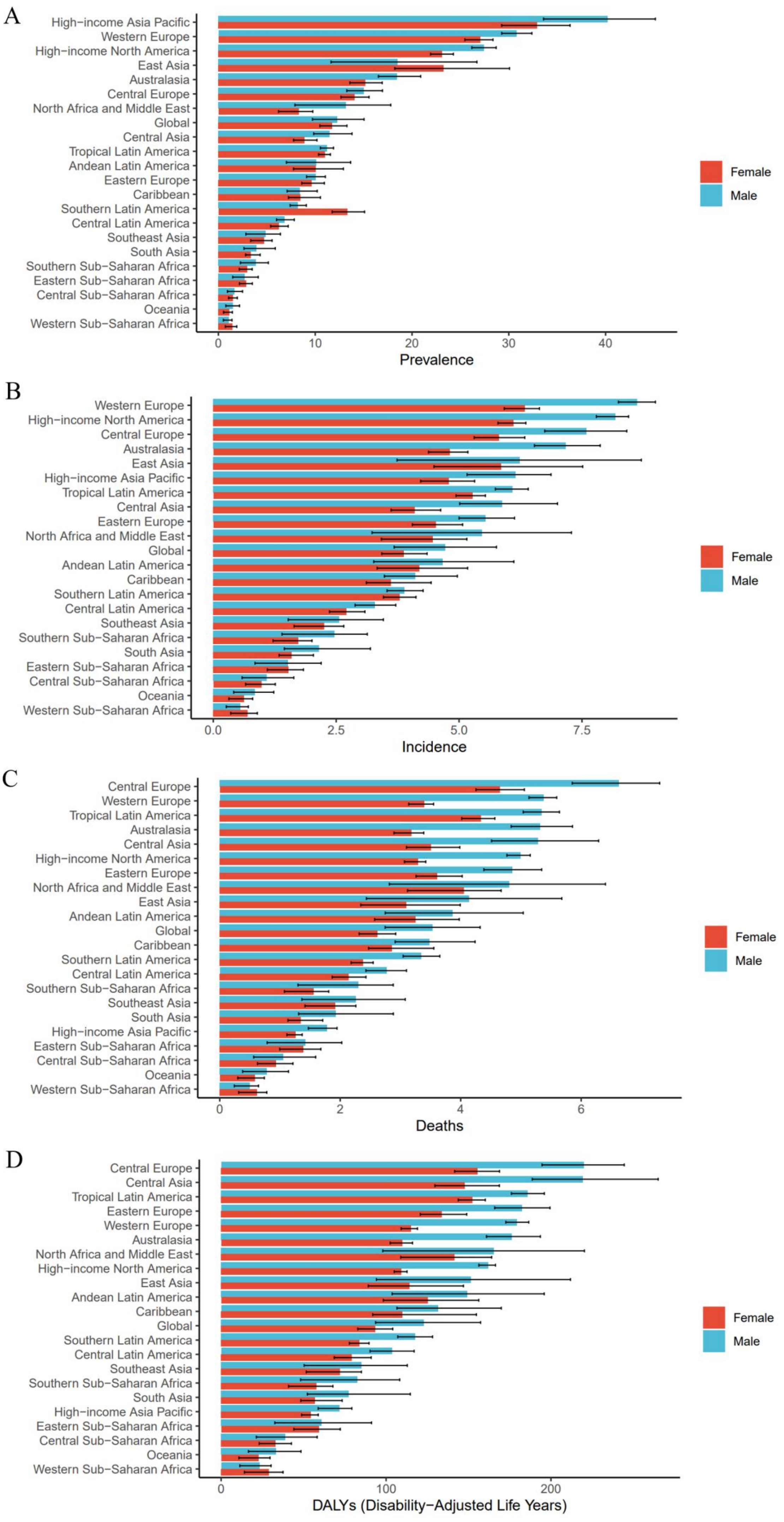

Supplementary Fig. 10 Comparison of the ASPR (A), ASIR (B), ASDR (C), and D for male and female Brain and CNS cancer across 21 global regions in 2021.
