# Supplementary material for: The global, regional, and national brain and central nervous system cancer burden and trends from 1990 to 2021: an analysis based on the Global Burden of Disease Study 2021
Source: Front Neurol. 2025 Jun 18;16:1574614. doi: 10.3389/fneur.2025.1574614 (PMC12213423; doi:10.3389/fneur.2025.1574614)
Supplement: Supplementary file 1 [file Data_Sheet_1.zip › Supplementary Data/Supplementary Fig. 15.pdf]

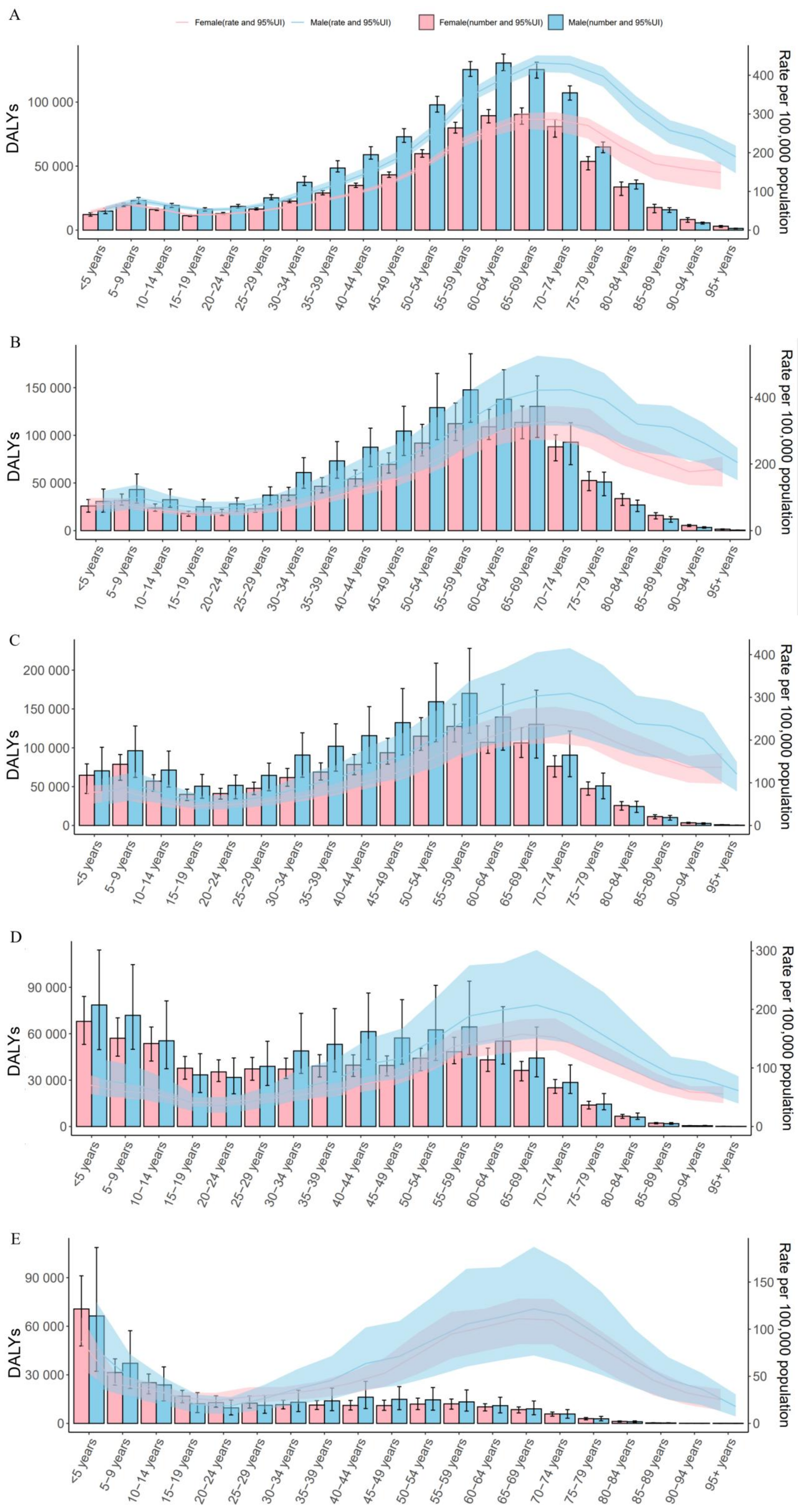

Supplementary Fig. 15 Regional trends in the number and rate of Brain and CNS cancer DALYs by age and sex in 2021.A.High SDI; B. High-middle SDI; C. middle SDI; D. Low-middle SDI; E. Low SDI.
