# Supplementary material for: The global, regional, and national brain and central nervous system cancer burden and trends from 1990 to 2021: an analysis based on the Global Burden of Disease Study 2021
Source: Front Neurol. 2025 Jun 18;16:1574614. doi: 10.3389/fneur.2025.1574614 (PMC12213423; doi:10.3389/fneur.2025.1574614)
Supplement: Supplementary file 1 [file Data_Sheet_1.zip › Supplementary Data/Supplementary Fig. 9.pdf]

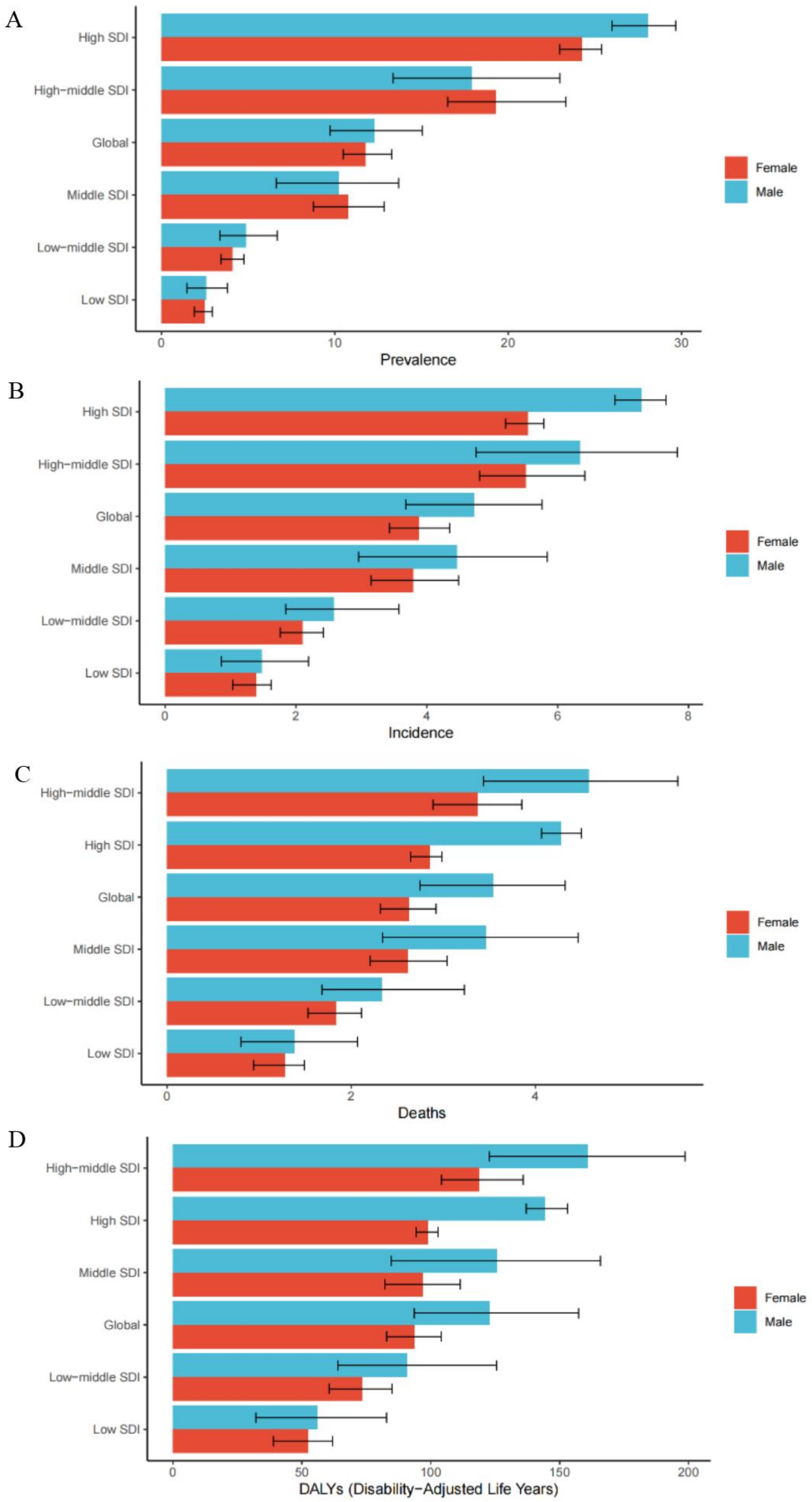

Supplementary Fig. 9 Comparison of the ASPR, ASIR, ASDR, and DALY rate for male and female Brain and CNS cancer across the five SDI regions globally in 2021.
A. ASPR; B. ASIR; C. ASDR; D. DALY rate.
