# Supplementary figures and images for: The global, regional, and national brain and central nervous system cancer burden and trends from 1990 to 2021: an analysis based on the Global Burden of Disease Study 2021
Source: Front Neurol. 2025 Jun 18;16:1574614. doi: 10.3389/fneur.2025.1574614 (PMC12213423; doi:10.3389/fneur.2025.1574614)

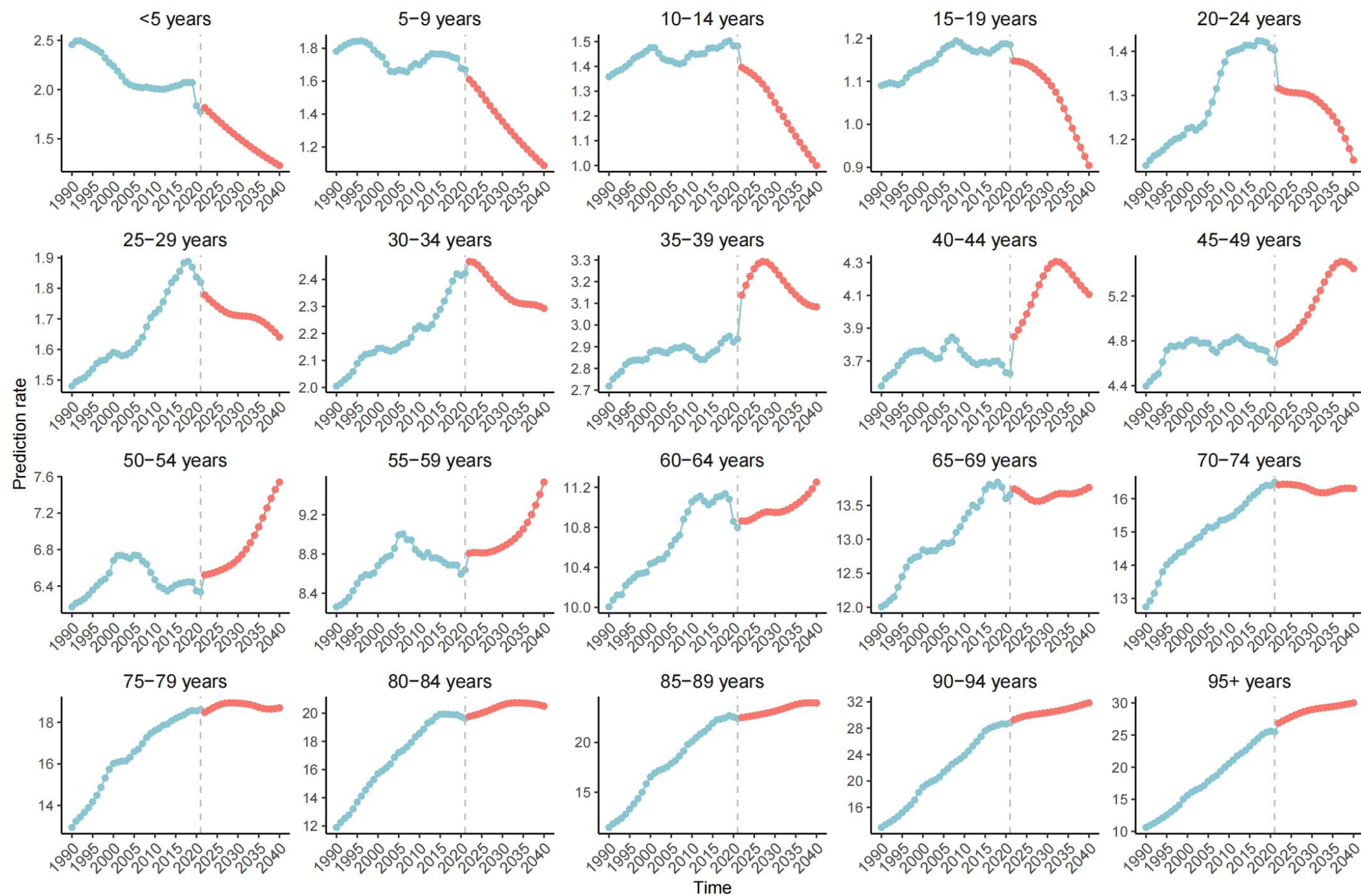

**Supplementary Fig. 17** Projections for the ASIR of Brain and CNS cancer by 2040.

Supplement: Supplementary file 1 [file Data_Sheet_1.zip › Supplementary Data/Supplementary Fig. 17.pdf]

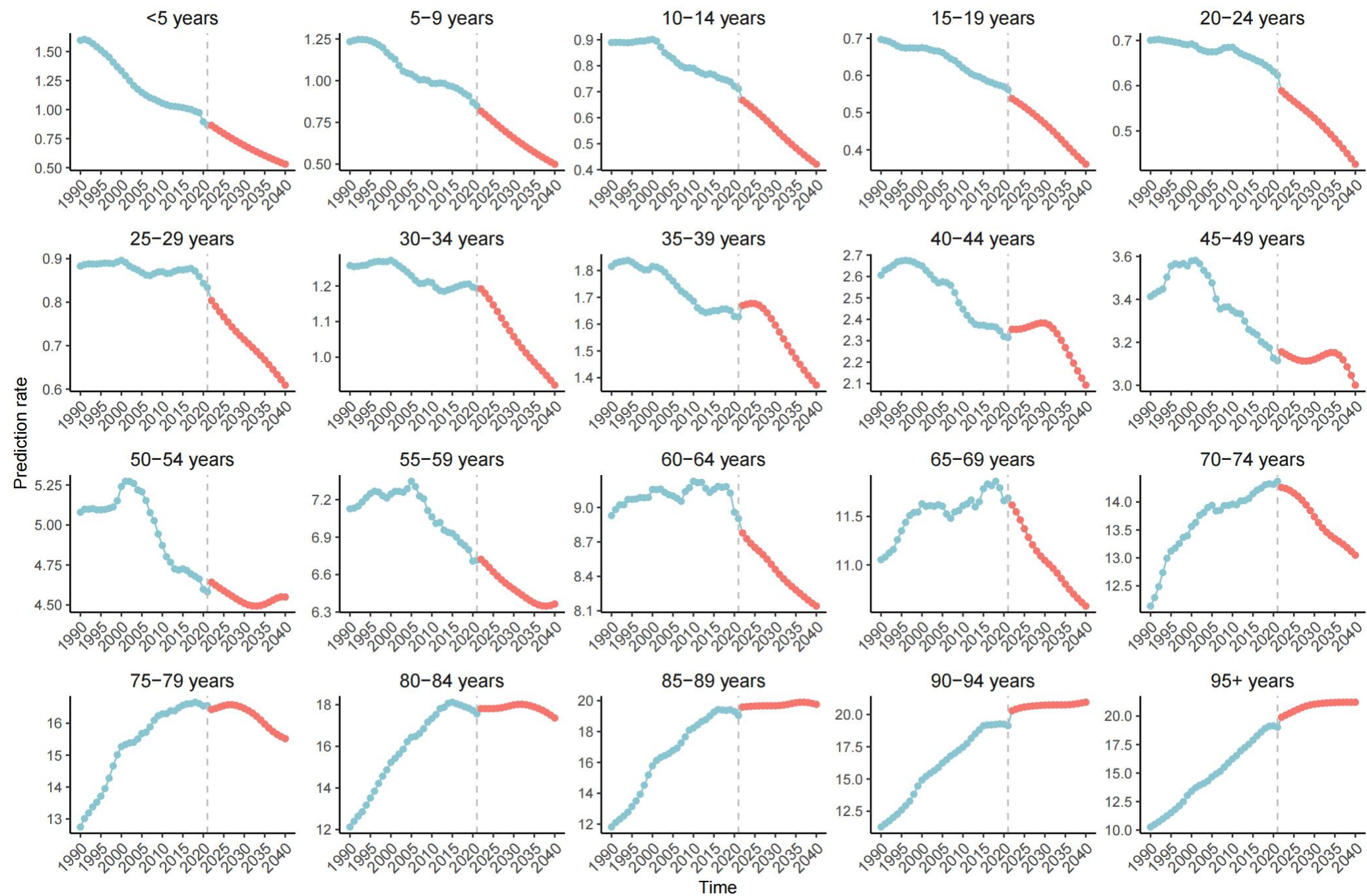

**Supplementary Fig. 18** Projections for the ASDR of Brain and CNS cancer by 2040.

Supplement: Supplementary file 1 [file Data_Sheet_1.zip › Supplementary Data/Supplementary Fig. 18.pdf]

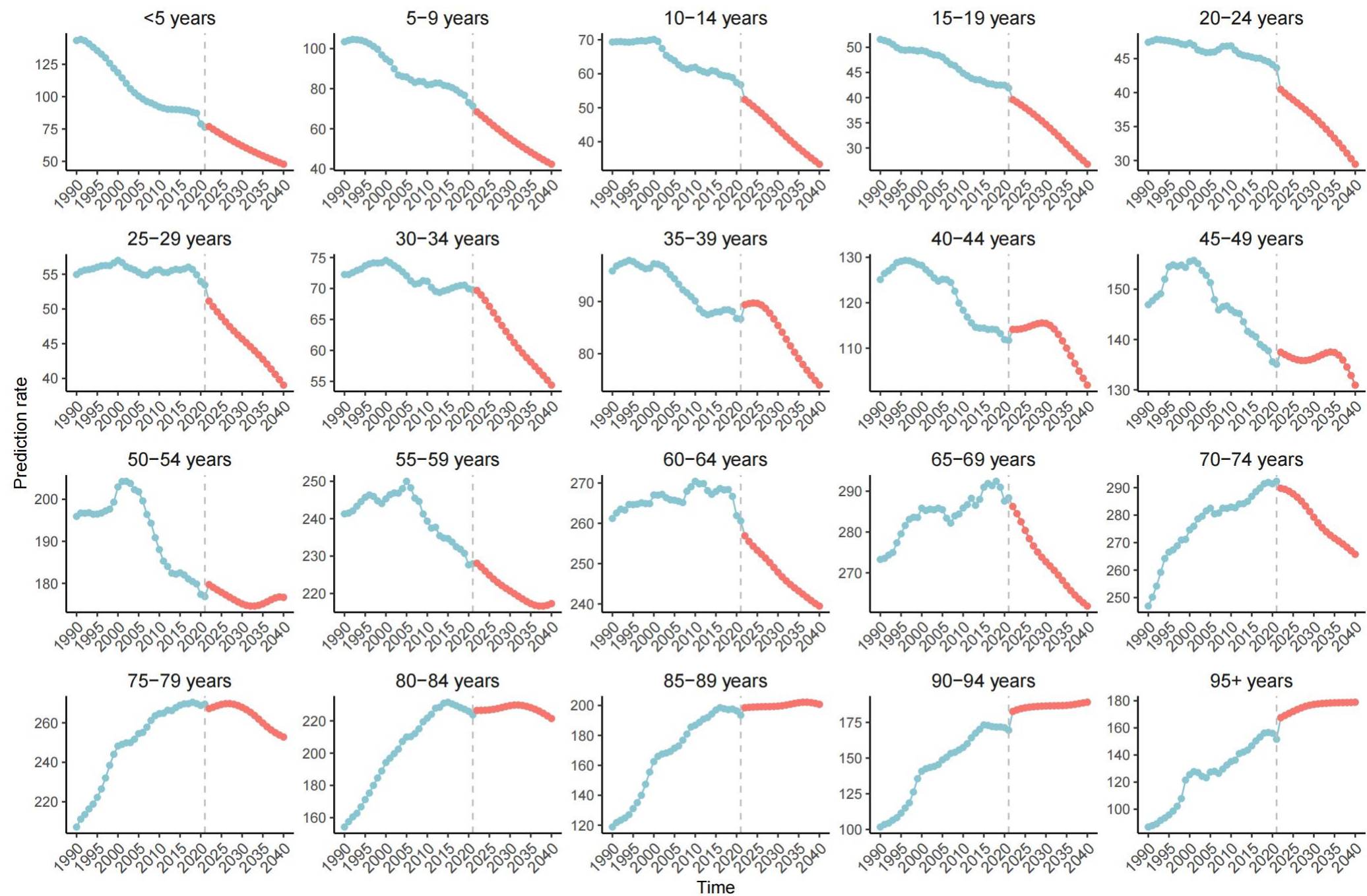

**Supplementary Fig. 19** Projections for the age-standardized DALY rate of Brain and CNS cancer by 2040.

Supplement: Supplementary file 1 [file Data_Sheet_1.zip › Supplementary Data/Supplementary Fig. 19.pdf]
